# Maternal and Early Neonatal Serum Cytokine and Endothelin-1 Concentrations in Preeclampsia: A Single-Center Observational Cohort Study

**DOI:** 10.3390/children13081039

**Published:** 2026-08-04

**Authors:** Christos-Georgios Kontovazainitis, Dimitra Gialamprinou, Alexandra Fleva, Anastasia Giannakou, Maria-Elina Bessina, Maria Varsami, Theodoros Theodoridis, Christina Mitsiakou, Elissavet Diamanti, Georgios Mitsiakos

**Affiliations:** 1 2nd Neonatal Department and Neonatal Intensive Care Unit (NICU), “Papageorgiou” University Hospital, Aristotle University of Thessaloniki, 56403 Thessaloniki, Greece; chgkontovazainitis@gmail.com (C.-G.K.); gialamprinou@gmail.com (D.G.); diamanti.veta@gmail.com (E.D.); 2 Immunology and Histocompatibility Department, “Papageorgiou” University Hospital, 56403 Thessaloniki, Greece; alexfleva@gmail.com (A.F.); giannakouan@gmail.com (A.G.); manina1975@gmail.com (M.-E.B.); mikroli31@gmail.com (M.V.); 3 1st Department of Obstetrics and Gynecology, “Papageorgiou” University Hospital, Aristotle University of Thessaloniki, 56403 Thessaloniki, Greece; theodtheo@yahoo.gr; 4 2nd Department of Paediatrics, “AHEPA” University Hospital, Aristotle University of Thessaloniki, 54636 Thessaloniki, Greece; christina.mits@outlook.com

**Keywords:** preeclampsia, cytokines, interleukins, IL2, IL6, IL8, TNFα, endothelin, ET1, neonatal complications

## Abstract

**Highlights:**

**What are the main findings?**

- After false discovery rate (FDR) correction, maternal endothelin 1 (ET1) was higher in women with preeclampsia (PE), and neonatal interleukin 2 (IL2) and tumor necrosis factor α (TNFα) were higher in PE-exposed neonates; after gestational-age at birth adjustment, the neonatal TNFα difference persisted, whereas IL2 was attenuated.
- None of the exploratory Firth biomarker–outcome associations remained significant after FDR correction; thrombocytopenia at birth (5/33 vs. 1/47) and intraventricular hemorrhage (IVH) (2/34 vs. 2/47) were retained as descriptive clinical context.

**What are the implications of the main findings?**

- Concurrent maternal and early neonatal sampling identified between-group differences in inflammatory and endothelial biomarkers associated with PE at delivery, with maternal ET1 and neonatal TNFα as the most robust signals.
- The complication-related findings are hypothesis-generating and do not support predictive cut-offs, biomarker-based neonatal risk stratification, or causal mechanisms.

**Abstract:**

**Background/Objectives:** Preeclampsia (PE) may be associated with maternal endothelial activation and altered early neonatal inflammation. The primary objective was to compare maternal and early neonatal serum interleukin 2 (IL2), IL6, IL8, tumor necrosis factor α (TNFα), and endothelin 1 (ET1) concentrations between PE and control mother–neonate pairs. **Methods:** This secondary report from a single-center observational cohort included 31 women with PE (34 neonates) and 45 control women (47 neonates). Biomarker concentrations were log-transformed, and PE–control differences were estimated using linear models, with results expressed as geometric mean ratios (GMRs). Neonatal models used pregnancy-clustered standard errors to account for twins. The Benjamini–Hochberg procedure was applied to control the false discovery rate (FDR). Additional neonatal models adjusted for gestational age at birth served as sensitivity analyses. Selected biomarker–outcome associations were explored post hoc using univariable Firth bias-reduced logistic regression. **Results:** Maternal ET1 was higher in PE (GMR = 1.79, 95% confidence interval—CI—1.53–2.09; q < 0.001). Neonatal IL2 (GMR = 1.37, 95% CI 1.11–1.71; q = 0.016) and TNFα (GMR = 1.49, 95% CI 1.22–1.82; q < 0.001) were higher in PE-exposed neonates. After gestational age at birth adjustment, the TNFα difference persisted (GMR = 1.33, 95% CI 1.14–1.55; q = 0.003), whereas IL2 was attenuated (GMR = 1.20, 95% CI 0.99–1.45; q = 0.164). None of the exploratory Firth associations remained significant after FDR correction. Thrombocytopenia at birth occurred in 5/33 PE-exposed and 1/47 control neonates; intraventricular hemorrhage occurred in 2/34 and 2/47, respectively. **Conclusions:** PE was associated with between-group differences in maternal endothelial and early neonatal inflammatory biomarkers, with maternal ET1 and neonatal TNFα as the most robust signals. Complication-related findings remain hypothesis-generating and do not support predictive cut-offs, risk stratification, or causal inference.

## 1. Introduction

Preeclampsia (PE), estimated to affect 3–8% of pregnancies worldwide, remains a major contributor to maternal and perinatal morbidity [1,2]. Beyond maternal disease, PE is linked with fetal growth restriction, oligohydramnios, placental abruption, prematurity, neonatal thrombocytopenia, bronchopulmonary dysplasia (BPD), and necrotizing enterocolitis (NEC) [1,2,3,4]. These neonatal complications are likely multifactorial, reflecting the interaction between prematurity, placental dysfunction, fetal hypoxia, and postnatal exposures rather than a single pathogenic pathway.

The pathogenesis of PE is rooted in abnormal early placentation and endothelial dysfunction, marked by endothelin-1 (ET1) overexpression and mediated by interleukins 2, 6, and 8 (IL2, IL6, and IL8) and tumor necrosis factor-α (TNFα) [5,6]. Maternal and fetal cytokine compartments are not directly interchangeable because transplacental transfer appears to be cytokine-specific, and evidence from ex vivo human placental perfusion studies is inconsistent. One study demonstrated bidirectional transfer of IL6 and minimal maternal-to-fetal transfer of TNFα, whereas another detected no transfer of IL6, TNFα, or interleukin 1β across normal-term placenta [7,8]. Neonatal cytokine concentrations may therefore reflect selective transfer and/or placental or fetal inflammatory responses rather than simple passive diffusion; cord-blood evidence nevertheless supports fetal endothelial activation in pregnancies complicated by PE [9]. Independently of PE, raised inflammatory mediators measured in umbilical blood, amniotic fluid, or other compartments, especially IL6, IL8, and TNFα, are associated with neonatal infection, inflammatory syndromes [10,11,12], and complications such as respiratory distress syndrome, early-onset sepsis, and intraventricular hemorrhage [13]. However, no single cytokine has adequate sensitivity and specificity for prediction or diagnosis [14,15], and evidence for IL2 remains limited [16,17].

The primary objective of the present report was to compare maternal and early neonatal serum concentrations of ET1, IL2, IL6, IL8, and TNFα between PE and control mother–neonate pairs. Concurrent maternal and neonatal sampling was used to characterize the inflammatory and endothelial biomarker differences associated with PE at delivery. Associations with severe PE, respiratory distress syndrome (RDS), and neonatal thrombocytopenia were not prespecified and were evaluated post hoc using exploratory univariable analyses within the PE-exposed groups. Maternal platelet transfusion, maternal postpartum thrombocytopenia, BPD, intraventricular hemorrhage (IVH), NEC, and retinopathy of prematurity (ROP) were summarized descriptively because of their small event counts. Accordingly, all post hoc complication-related findings were interpreted as hypothesis-generating rather than predictive.

## 2. Materials and Methods

This article is a secondary report from a previously established observational cohort described in Kontovazainitis et al. [18]. The original protocol encompassed three related components: (i) between-group comparisons of Rotational Thromboelastometry (ROTEM) parameters; (ii) between-group comparisons of maternal and neonatal circulating biomarkers; and (iii) analyses of the relationships between biomarker concentrations and ROTEM parameters. Because these questions were distinct, the findings were divided into separate reports, and the ROTEM comparisons have been published previously [18]. This study focuses primarily on the protocol-specified comparison of maternal and neonatal serum IL2, IL6, IL8, TNFα, and ET1 concentrations between the PE and control groups. Associations of biomarkers with PE severity, transfusion, and neonatal morbidities were not prespecified and were explored after data collection. More specifically, after completion of these protocol-specified comparisons, we conducted secondary post hoc analyses exploring associations between the measured biomarkers and PE severity, maternal platelet transfusion, and selected neonatal complications, including thrombocytopenia, BPD, NEC, IVH, ROP, and RDS.

None of the endpoints reported in this article were included in the previous publication; the present report also includes statistical analyses specific to these endpoints. Because outcome-specific analyses were performed in a limited number of exposed neonates, they were considered exploratory.

### 2.1. Study Design, Setting, and Participants

This single-center observational study assessed IL2, IL6, IL8, TNFα, and ET1 serum levels in pregnant women with PE and their neonates and compared them with those of healthy mother–neonate pairs at the “Papageorgiou” Hospital (March 2022–March 2024). The study descriptively characterized the listed neonatal morbidities; selected biomarker–outcome associations were explored post hoc as specified below. Thus, this study evaluated the association between maternal and neonatal cytokine and ET1 levels among PE-exposed neonates with the following neonatal complications: neonatal thrombocytopenia at birth (<150 × 10^9^/L), BPD, IVH, NEC, ROP, and RDS. Neonatal morbidities were collected during follow-up for descriptive characterization rather than biomarker-based prediction.

The study adhered to the Declaration of Helsinki and was approved by the Aristotle University of Thessaloniki Department of Bioethics (No. 48/202, 14 February 2022) and the Institutional Review Committee (No. 20192/2022, 4 February 2022). Written informed consent was obtained from all participants, including consent for the collection and use of neonatal clinical data and biologic samples. The study enrolled women who provided informed consent for themselves and their offspring. One participant, who had been recorded as being 18 years old at enrollment, was subsequently found during verification of the study records to have been 17 years old when the original consent form was signed. That form covered the clinical data and biological samples of both the mother and her neonate. This protocol deviation and the proposed corrective procedure were reported in writing to the Aristotle University of Thessaloniki Department of Bioethics, and a written authorization was obtained. After reaching adulthood, the participant provided renewed written informed consent in person on 16 October 2024, when she was 19 years old, authorizing the continued processing and research use of both her own and her neonate’s data and samples. The mother–neonate pair was retained only following this documented ethical review and renewed consent. The approved protocol explicitly permitted preliminary eligibility screening using routinely collected clinical records before formal enrollment and written informed consent. Potential participants were invited sequentially during the study period according to the contemporaneous eligibility and delivery-proximity procedure described below; no propensity-score model was fitted during recruitment. Women who declined participation or subsequently withdrew consent for maternal and neonatal participation were excluded from the final analytic cohort.

**Figure 1:**
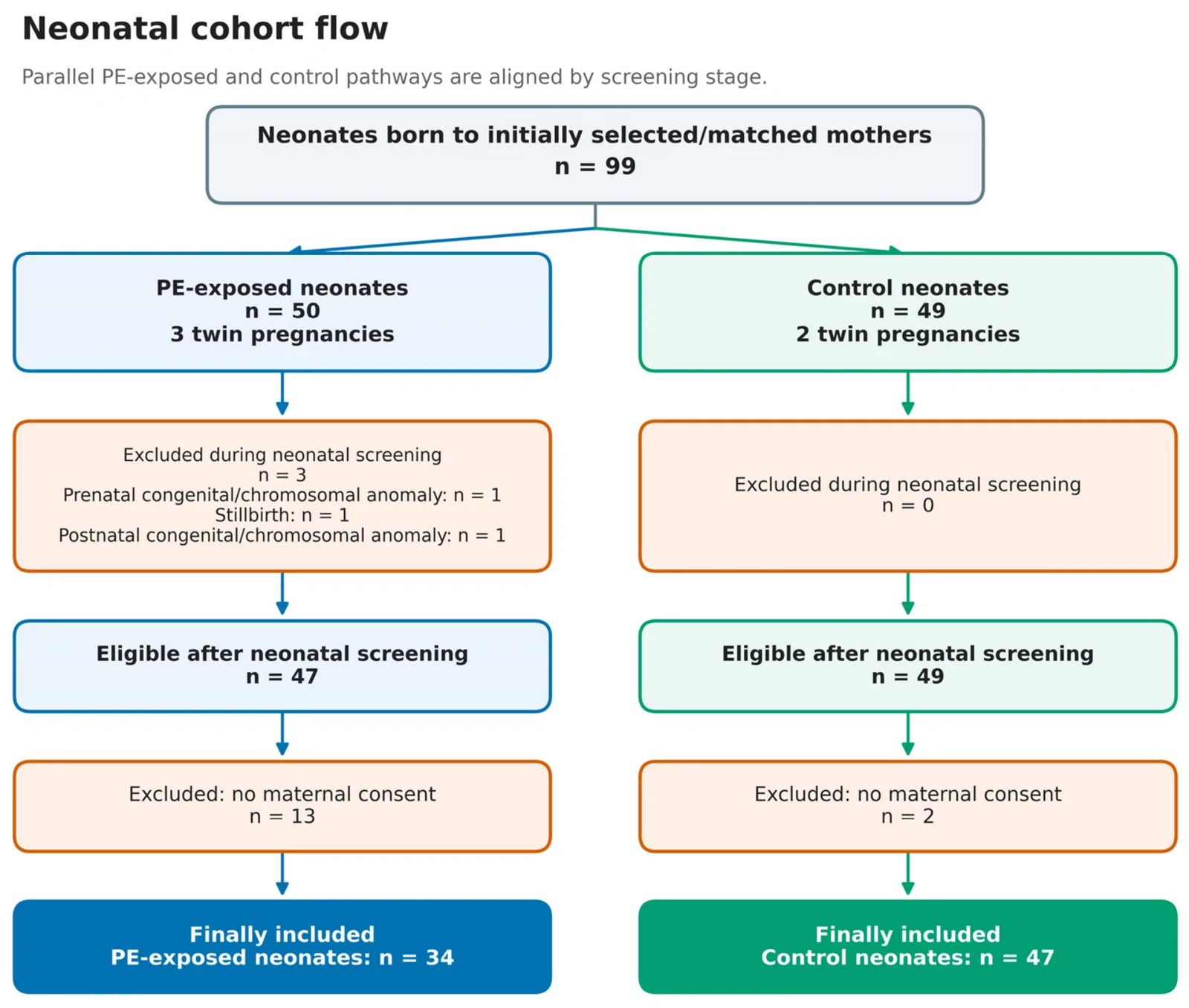
Study flow diagram of the neonatal cohort. Parallel PE-exposed and control pathways are presented from the neonates born to the initially selected/matched mothers through neonatal screening and final inclusion. The number of twin pregnancies in each group is shown. Abbreviation: PE, preeclampsia.

**Figure 2:**
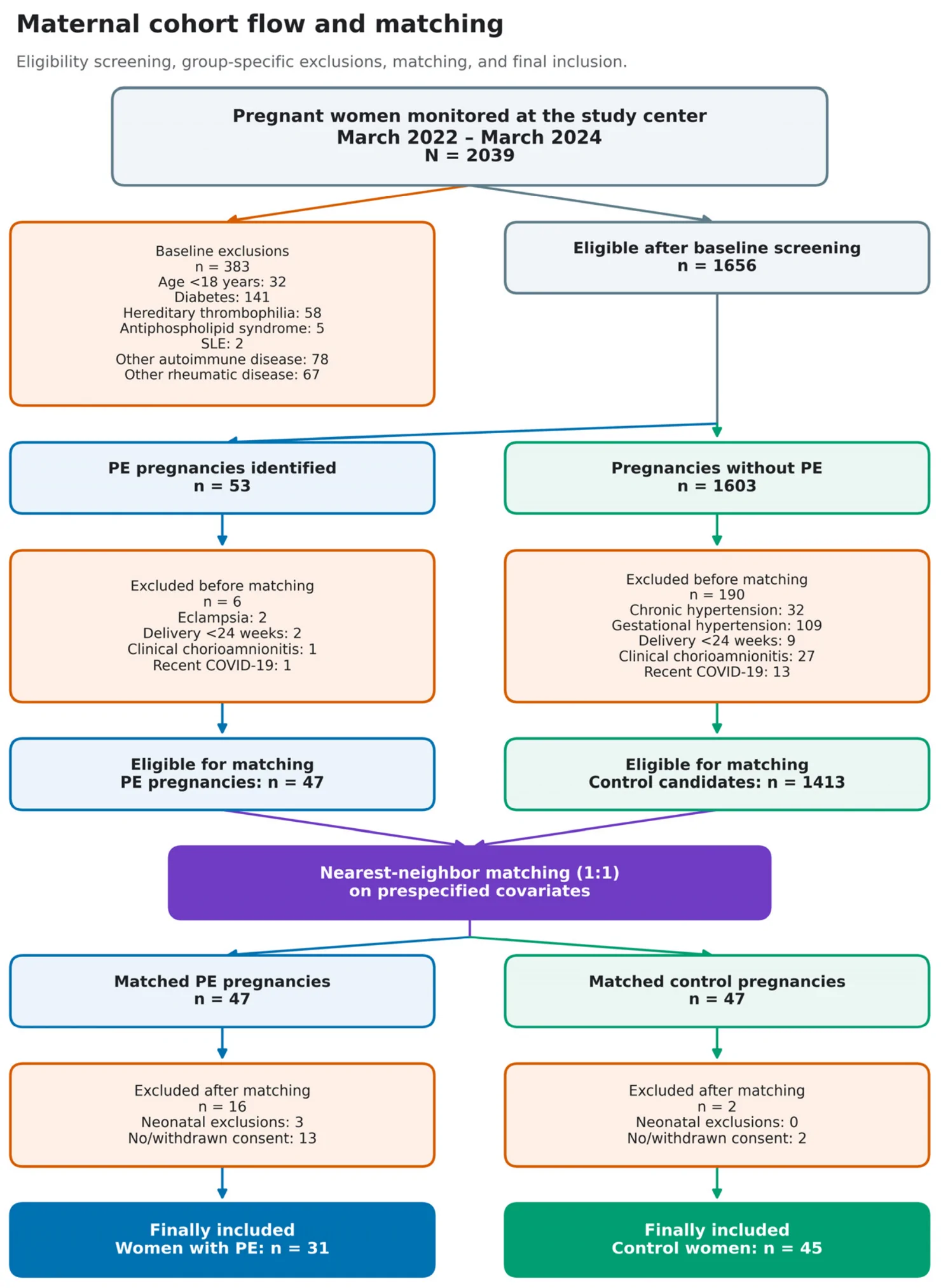
Study flow diagram of the maternal cohort and matching process. The diagram presents baseline eligibility screening, group-specific exclusions, 1:1 nearest-neighbor matching, post-matching exclusions, and final maternal inclusion. The matching stages represent the retrospective analytical construction of the cohort and not the chronological sequence of recruitment, consent withdrawal, biospecimen collection, and model fitting. Abbreviations: COVID-19, coronavirus disease 2019; PE, preeclampsia; SLE, systemic lupus erythematosus.

The study’s inclusion and exclusion criteria are discussed elsewhere [18]. Eligibility was assessed using the identification documentation available at recruitment. Pregnant women were eligible if they were documented as aged ≥ 18 years, with gestational age (GA) ≥ 20 + 0 weeks at enrollment and GA ≥ 24 + 0 weeks at delivery. One participant met the age-eligibility criterion according to the documentation available at recruitment; official verification during subsequent verification of the study records after enrollment established that she was 17 years old. The corrected age was retained in the dataset and is reported transparently. We excluded women with chronic hypertension before 20 gestational weeks (GW), gestational hypertension, diabetes mellitus, hereditary thrombophilias, antiphospholipid syndrome, systemic lupus erythematosus, other autoimmune or rheumatic disease, coronavirus disease 2019 (COVID-19) within 15 days prior to delivery, or clinical chorioamnionitis. Women in the control group met the same eligibility criteria but did not have PE or another hypertensive disorder of pregnancy. For the neonatal component, all liveborn infants of enrolled mothers were assessed for eligibility; exclusions were stillbirth, perinatal asphyxia, and any prenatal or postnatal diagnosis of chromosomal abnormalities or other congenital anomalies. Exclusion of an infant led to exclusion of the mother (mother–infant pair) (Figure 1 and Figure 2). GA at recruitment referred to gestational age at the initial eligibility assessment using routine antenatal records; it did not represent GA at PE diagnosis or delivery. Women who remained eligible were followed through delivery. Those who developed PE entered the PE candidate pool, whereas women who completed pregnancy without PE or another hypertensive disorder remained potential control candidates. Each woman was counted once according to the first applicable exclusion criterion; the exclusion categories in Figure 2 were mutually exclusive.

Demographic characteristics and medical history were collected for each mother–neonate pair from medical records and structured interviews, and participants were followed until hospital discharge.

### 2.2. Definitions

Gestational hypertension, PE, PE with severe features, and hemolysis, elevated liver enzymes, and low platelet count (HELLP) syndrome were defined according to the American College of Obstetrics and Gynecology (ACOG) criteria [6,19]. More precisely, gestational hypertension was defined as blood pressure ≥ 140/90 mmHg on two occasions at least 4 h apart after 20 GW in a woman with previously normal blood pressure. PE was defined as new-onset hypertension (blood pressure ≥ 140/90 mmHg on two occasions at least 4 h apart in a previously normotensive woman) after 20 GW, accompanied by either proteinuria or, in the absence of proteinuria, new-onset maternal end-organ dysfunction. Proteinuria was defined as ≥300 mg of protein in a 24-h urine collection. In accordance with the ACOG criteria, end-organ dysfunction included thrombocytopenia (platelet count < 100 × 10^9^/L), renal insufficiency (serum creatinine > 1.1 mg/dL or a doubling of the baseline value), impaired liver function (elevated liver transaminases to ≥2 times the upper limit of normal), pulmonary edema, or new-onset cerebral or visual symptoms. PE with severe features, hereafter referred to as severe PE, was defined by severe-range hypertension (systolic blood pressure ≥ 160 mmHg or diastolic blood pressure ≥ 110 mmHg) or by any of the aforementioned ACOG end-organ dysfunction criteria. PE without severe features, hereafter referred to as non-severe PE, was defined as PE in the absence of any severe feature [6,19]. The National Institute for Health and Care Excellence (NICE) guidelines were also considered; we used the presence of uteroplacental dysfunction as a diagnostic criterion (fetal growth restriction or abnormal umbilical artery Doppler findings in the presence of hypertension), and thrombocytopenia was defined as a platelet count (PLT) < 150 × 10^9^ [20]. The NICE platelet threshold of <150 × 10^9^/L was not used in isolation to classify PE as severe. In the present cohort, all women with PLT counts between 100 and 149 × 10^9^/L who were classified as having severe PE had at least one additional ACOG severe feature. Early-onset PE was defined as PE occurring before 34 GW [2,21,22]. HELLP syndrome was defined as lactate dehydrogenase (LDH) ≥ 600 IU/L, aspartate aminotransferase (AST) and alanine aminotransferase (ALT) ≥ 2 times the upper normal limit, and PLT count < 100 × 10^9^/L. Posterior reversible encephalopathy syndrome (PRES) was identified from the medical record when compatible acute neurological manifestations were supported by characteristic neuroimaging findings. No cases of PRES were recorded in the present cohort. Chronic hypertension was defined based on the ACOG’s criteria (hypertension diagnosed or present before pregnancy or before 20 weeks of gestation) [19]. Labor management was categorized as pharmacological induction using misoprostol and oxytocin or amniotomy (artificial rupture of membranes).

Neonatal outcomes were assessed from birth until hospital discharge and were coded as binary variables (present/absent) for the present analysis.

Neonatal thrombocytopenia at birth was defined as a PLT count less than 150 × 10^9^/L in the neonatal arterial blood sample obtained within the first hour of life [23,24].

BPD was defined based on Higgins et al. [25]. BPD was assessed among neonates born at <32 GW. According to the standard practice of the neonatal unit, these neonates were screened for evolving BPD on postnatal day 28, and BPD status and severity grade were determined at 36^+0^ weeks’ postmenstrual age (PMA) according to the 2018 Eunice Kennedy Shriver National Institute of Child Health and Human Development (NICHD) workshop criteria. All surviving eligible neonates born at <32 GW in the present cohort remained hospitalized through 36^+0^ weeks’ PMA and underwent the scheduled assessment. BPD was clinically classified into grades I–III based on the required mode of respiratory support and oxygen concentration. For the present analysis, BPD was coded as present or absent, and severity grades were not analyzed.

All enrolled neonates underwent at least one cranial ultrasonographic examination within the first 24 h of life as part of the study assessment and a concurrent neonatal protocol. Neonates born at <32 GW or with a birthweight < 1500 g underwent routine serial cranial ultrasonography on postnatal days 1, 3, and 7. Additional examinations were performed when the initial examination was abnormal or when clinically indicated. IVH was graded from I to IV according to the Papile classification and the criteria described by Volpe et al. [26,27,28]. Any IVH grade was coded as IVH for the present analysis; individual grades were not analyzed. The individual grades were reported descriptively.

NEC was assessed throughout hospitalization and was defined by compatible clinical and radiographic findings corresponding to modified Bell stage II or higher [29,30]. Suspected NEC corresponding to stage I was not classified as NEC. NEC was analyzed as a binary outcome.

All preterm neonates born at <32 GW or with a birthweight < 1500 g underwent their first ROP screening examination between postnatal days 21 and 28. Screening was performed by a pediatric ophthalmologist using dilated binocular indirect ophthalmoscopy, with subsequent examinations scheduled according to retinal findings. ROP was classified by zone, stage, and the presence or absence of plus disease according to the 3rd International Classification of ROP [31,32]. For the present analysis, any ROP stage was coded as ROP, and individual stages were not analyzed.

Neonatal RDS was defined based on the 2022 European Consensus Guidelines [33]. RDS was assessed immediately after birth and was defined by the early onset of clinical respiratory distress requiring supplemental oxygen or respiratory support, supported—where available—by chest radiographic or lung-ultrasound findings compatible with surfactant deficiency and after exclusion of alternative causes. RDS was analyzed as a binary outcome, without severity grading.

### 2.3. Sample Collection and Laboratory Analyses

Maternal specimens consisted of venous whole blood, whereas neonatal specimens were obtained from arterial blood. Maternal blood was collected before placental delivery: during labor in women who delivered vaginally and before transfer to the operating room in women who underwent cesarean delivery. The timing of maternal sampling relative to the administration of magnesium sulfate, antihypertensive medications, and antenatal corticosteroids was not standardized by the study protocol and depended on routine clinical care. Neonatal specimens consisted of peripheral arterial blood obtained from the radial artery as part of the first postnatal blood draw, within the first hour of life. Cord blood was not used. Because neonatal sampling was integrated into routine clinical care, its timing relative to initial resuscitation and the initiation of oxygen or respiratory support was not protocol-standardized.

For each participant, samples were drawn into one tripotassium ethylenediaminetetraacetic acid (K3-EDTA) tube, one trisodium citrate tube (0.109 mol/L), and two serum separator tubes and were processed within 60 min. Hematological indices were measured using the Alinity-hq analyzer, and biochemical parameters were measured using the Alinity-c system (Abbott GmbH, Wiesbaden, Germany). Antithrombin III, Protein C, and Protein S were determined with STA-Stachrom-ATIII, STA-Stachrom-ProteinC, and STA-Liatest-FreeProteinS reagents, respectively, on the STAR-MAX analyzer (Diagnostica Stago S.A.S., Asnières-sur-Seine, France).

Regarding cytokine and ET1 measurements, for each sample, one serum separator tube was centrifuged for 15 min, and then 600 μL of clear serum was collected and equally divided into two Eppendorf tubes. Serum aliquots were stored at −70 °C for no longer than six months before analysis. Samples were analyzed in batches after a single freeze–thaw cycle. Each biomarker was measured in a single determination. All specimens were identified solely by study codes, and laboratory personnel were blinded to maternal group allocation and clinical outcomes.

IL2, IL6, IL8, and TNFα were measured according to the manufacturer’s protocol by flow cytometry (8-color Navios, Beckman Coulter, Miami, FL, USA) using Human Custom 4-Plex premixed bead-based immunoassay kits (AimPlex Biosciences, Inc., Pomona, CA, USA; catalog no. T1C0421209). Three identical 96-well kits of the same product configuration were used to analyze 157 coded serum specimens across seven measurement cycles, with a maximum of 25 specimens per cycle (cycle 1: 25, cycle 2: 25, cycle 3: 25, cycle 4: 25, cycle 5: 25, cycle 6: 25, cycle 7: 7). All measurements were performed in the same laboratory using the same flow cytometer, laboratory team, and analytical protocol. Each specimen was measured in a single determination, whereas the calibration standards and blank were analyzed in duplicate ([8 + 1] × 2 = 18 wells per cycle). For each analyte, a common calibration function was generated from the combined and averaged standard readings retained across the measurement cycles and was applied to all specimens. Median fluorescence intensity values were converted to concentrations using FlowJo software, v10.10. The retained Standard Information Sheet identified the lyophilized Standard Mix as part number T1C0421209S, Lot 4012531. The manufacturer-reported limits of detection for IL2, IL6, IL8, and TNFα were <3, <5, <1, and <1 pg/mL, respectively. The corresponding lower limits of quantification were <10, <10, <1, and <2 pg/mL, and the upper limits of quantification were >2000, >5000, >1000, and >1000 pg/mL. The manufacturer also reported a standard-dose recovery of 70–130%, an intra-assay coefficient of variation < 10%, and an inter-assay coefficient of variation < 20%. These analytical limits were reported by the manufacturer as inequality bounds. Exact lot-specific numerical limit of detection (LOD) and lower limit of quantification (LLOQ) values were not provided in the retained documentation. FlowJo-derived concentrations were retained without fixed-value substitution. Measurements below the lowest non-zero calibration standard were treated as non-quantifiable and excluded from all analyses; no fixed-value substitution or imputation was applied.

ET1 was measured according to the manufacturer’s instructions with an enzyme-linked immunosorbent assay (ELISA) using the Human Endothelin-1 QuantiGlo chemiluminescent ELISA Kit (QET00B, R&D SYSTEMS, Minneapolis, MN, USA), with a reported analytical sensitivity of 0.102 pg/mL, a measurement range of 0.34–250 pg/mL, an intra-assay coefficient of variation of 2.6–3.4%, and an inter-assay coefficient of variation of 4.5–9.1%.

### 2.4. Strategies to Address Bias and Within-Pregnancy Dependence

To reduce measured baseline imbalance, 47 eligible PE pregnancies were matched 1:1 to 47 pregnancies selected from 1413 eligible control candidates using propensity scores estimated by logistic regression. The matching variables were maternal age, GA at recruitment, pre-pregnancy body mass index, maternal age ≥ 40 years, PE in a previous pregnancy, aspirin use, low-molecular-weight heparin use, progesterone use, and antenatal corticosteroid administration. During the study period, controls were recruited sequentially to permit contemporaneous sampling. When a PE pregnancy approached delivery, eligible control candidates who were also approaching delivery were invited to participate. This operationally sequential recruitment procedure was distinct from the formal propensity-score matching. After recruitment had been completed, a single global propensity-score model was fitted retrospectively using the complete eligible candidate dataset. The 47 control records selected by this model corresponded to the controls who had been recruited. The retrospective model therefore formalized and confirmed the balance achieved through the operationally sequential recruitment procedure; it did not represent a separate second cohort-selection process. Baseline characteristics and medication exposures were coded using information documented before biospecimen collection. Antenatal corticosteroid exposure could occur after PE diagnosis, usually in anticipation of preterm delivery, and was therefore not regarded as a conventional pre-exposure confounder. It was included in the matching model to balance a pre-sampling treatment that could influence circulating biomarker concentrations. Accordingly, the resulting comparison should be interpreted as a design-conditioned PE–control biomarker contrast rather than as the unconditioned total effect of PE. Greedy nearest-neighbor matching was performed without replacement and without a caliper, with the PE group as the focal group and the average treatment effect among the treated (ATT) as the matching estimand; PE pregnancies were processed from the largest to the smallest propensity score (Table S1).

Covariate balance was evaluated using absolute standardized mean differences (SMDs) before matching, immediately after matching, and in the final analytic cohort. Immediately after matching, eight of nine variables had |SMD| ≤ 0.10, the remaining variable had |SMD| = 0.152, and no variable exceeded 0.20 (mean |SMD| = 0.052; maximum |SMD| = 0.152). After matching, 16 PE pregnancies (three neonatal exclusions and 13 women who declined or withdrew consent) and two control pregnancies (both declined or withdrew consent) were excluded, leaving 31 PE and 45 control pregnancies. When a PE pregnancy was excluded after matching, its initially matched control was retained if she remained eligible, maintained consent, and had valid samples; no post hoc rematching was performed. Consequently, the final cohort no longer retained the original 1:1 pair structure and was analyzed as two independent groups. In the final cohort, eight of the nine matching variables had |SMD| <0.20; the largest residual difference was for aspirin use (|SMD| = 0.308). These findings were not taken to establish complete balance (Table S1).

Information bias was mitigated through prespecified PE criteria, standardized clinical data collection, uniform sample collection and laboratory procedures, and laboratory blinding.

Potential confounding was addressed through retrospective propensity-score design procedure and the gestational-age-at-birth sensitivity analyses, while acknowledging residual confounding and possible selection bias from asymmetric postmatching exclusions. Baseline *p*-values were considered descriptive and were not used to assess balance or select adjustment covariates. The neonatal cohort included 34 neonates from 31 PE pregnancies and 47 neonates from 45 control pregnancies, corresponding to three and two twin pregnancies, respectively. Pregnancy was treated as the clustering unit in the neonatal biomarker models, and cluster-robust standard errors were used. The primary model estimand was the PE–control ratio of geometric mean biomarker concentrations in the final retained analytic cohort selected through matching and postmatching eligibility and consent; it was not interpreted as a population-average causal effect or as a formal ATT estimate after attrition. GA at birth was not included in the primary neonatal models because it may lie partly downstream of PE; secondary models adjusted for GA at birth as a sensitivity analysis and were interpreted as conditional associations rather than direct causal effects. Uterine artery pulsatility index was not included as an adjustment covariate because it represents a manifestation of the disease process. Unavailable measurements were reported separately for each group and analysis, no imputation was performed, and possible selection bias due to differential biomarker availability was considered during interpretation.

### 2.5. Sample Size

Sample-size planning was based on the primary comparison of biomarker concentrations between the PE and control groups and not on maternal or neonatal morbidity outcomes. Calculations assumed equal group allocation, 80% statistical power, a two-sided α level of 0.05, and an independent two-group comparison. Based on a previous study of maternal cytokines in women with PE [34], detecting the reported differences in IL6 and TNFα concentrations required two women per group because of the very large published effect sizes. Based on a study of maternal ET1 [35], detecting a mean difference of 98 pg/mL with a standard deviation (SD) of 64.2 pg/mL required eight women per group. Finally, based on a neonatal ET1 study [36], detecting a mean difference of 0.7 pg/mL with an SD of 1.18 pg/mL required 46 neonates per group. This was the largest estimated sample-size requirement among the biomarker comparisons considered and was therefore adopted as the recruitment target.

The original sample-size calculation did not account for multiple biomarker comparisons or the within-pregnancy correlation between twins. No post hoc power calculations were performed or used to interpret the observed findings. The study was not powered to assess associations with infrequent maternal or neonatal complications, perform multivariable prognostic modeling, or derive and validate clinically applicable biomarker cut-offs. Accordingly, the selected complication-related regression analyses were considered strictly post hoc and hypothesis-generating, whereas the remaining rare outcomes were summarized descriptively.

The final cohort comprised 31 women with PE and 45 control women, who delivered 34 and 47 neonates, respectively. Thus, the planned neonatal target of 46 participants per group was achieved in the control group but not in the PE-exposed group. This recruitment shortfall further limits the precision of the neonatal estimates and the interpretation of the exploratory outcome analyses.

### 2.6. Statistical Analysis

Original statistical analyses were conducted using RStudio version 2024.04.2, and revised analyses were conducted using RStudio version 2026.07.0. Baseline and clinical characteristics were summarized using the mean ± SD or median with interquartile range (IQR) for continuous variables and counts with percentages for categorical variables, as appropriate. Distributional assumptions were assessed separately for each continuous variable using histograms, normal quantile–quantile plots, and the Shapiro–Wilk test. Homogeneity of variance between groups was evaluated using Levene’s test. Continuous variables were compared using Student’s two-sample *t*-test when the distributional and equal-variance assumptions were considered reasonable, Welch’s *t*-test when the distributional assumption was reasonable but variances were unequal, and the Mann–Whitney U test when the distributional assumption was not supported. Categorical variables were assessed using the chi-squared test or Fisher’s exact test when expected cell counts were small. These exploratory comparisons of non-biomarker variables were considered descriptive and were not used to establish group comparability or to select adjustment covariates. For descriptive comparisons of continuous neonatal laboratory variables, *p*-values were adjusted for GA at birth and the 5-min Apgar score using Quade’s rank analysis of covariance (ANCOVA). Neonatal baseline comparisons were unadjusted. These analyses were also considered descriptive and were not included in the primary multiple-testing family.

For propensity-score diagnostics, SMDs were calculated for the nine matching variables in the pre-matching cohort (47 PE pregnancies and 1413 control candidates), the immediately matched cohort (47 and 47), and the final analytic cohort (31 and 45). Absolute SMDs, rather than hypothesis-test *p*-values, were used to evaluate covariate balance independently of sample size. More precisely, for the nine covariates included in the propensity-score model, balance diagnostics were calculated under the ATT estimand. Continuous-variable SMDs were standardized using the standard deviation of the PE group in the pre-matching cohort, whereas binary-variable SMDs were standardized using the corresponding pre-matching PE-group Bernoulli standard deviation. The same pre-matching standardization factors were retained before matching, immediately after matching, and in the final analytic cohort to permit direct comparison across stages.

Separately, the SMDs for maternal, neonatal and laboratory variables quantify descriptive between-group differences and were not interpreted as propensity-score matching diagnostics. For continuous variables, the absolute difference in group means was divided by the square root of the average of the two group variances. For binary variables, the absolute difference in proportions was divided by the square root of the average of the two Bernoulli variances. Continuous-variable SMDs were calculated from the raw observations, including for variables summarized as medians and interquartile ranges. These descriptive SMDs were unadjusted and were not used for statistical inference or covariate selection.

The primary analyses evaluated between-group differences in five maternal and five neonatal serum biomarkers: IL2, IL6, IL8, TNFα, and ET1. Because several biomarker distributions were right-skewed and all concentrations were positive, biomarker values were natural-log-transformed before model fitting. A separate linear model was fitted to the natural-log-transformed concentration of each biomarker, with PE exposure as the independent variable and the control group as the reference category. Model assumptions were evaluated using the residuals rather than the untransformed biomarker concentrations. Residual-versus-fitted plots and normal quantile–quantile plots were examined after transformation. Because PE exposure was binary in the primary models, no continuous-exposure linearity assumption was required. Maternal models used heteroscedasticity-consistent type 3 (HC3) standard errors to provide inference robust to unequal residual variances.

All neonates, including both members of twin pairs, were retained in the primary neonatal analyses. Pregnancy was specified as the clustering unit, and cluster-robust sandwich standard errors were calculated to account for both heteroscedasticity and within-pregnancy dependence. The same clustering approach was used in the gestational-age-adjusted neonatal sensitivity models. Model coefficients for PE exposure were exponentiated and are reported as geometric mean ratios (GMRs), each with a 95% confidence interval. A GMR > 1 indicates a higher geometric mean biomarker concentration in the PE group, whereas a GMR < 1 indicates a lower concentration. The active sample size and, for neonatal analyses, the number of contributing pregnancies are reported for every model [37]. Results for all five maternal and five neonatal biomarkers are presented irrespective of statistical significance.

The primary maternal and neonatal biomarker comparisons constituted a single multiple-testing family. The Benjamini–Hochberg procedure was applied to the corresponding model-derived *p*-values to control the false discovery rate, and both unadjusted *p*-values and false discovery rate (FDR)-adjusted q-values are reported [38]. Separately, the five gestational-age-adjusted neonatal sensitivity models constituted a secondary multiple-testing family and were independently corrected using the Benjamini–Hochberg procedure. The primary and sensitivity families were not combined because they addressed different estimands. Statistical interpretation focused primarily on effect estimates and their 95% confidence intervals. All tests were two-sided; *p* < 0.05 was considered nominally significant, whereas q < 0.05 indicated evidence remaining after the corresponding FDR correction.

The primary neonatal models estimated the PE–control contrast in the final analytic cohort following retrospective propensity-score design procedure on GA at recruitment and the other specified variables, but without regression adjustment for GA at birth. These estimates therefore describe the selected analytic cohort and should not be interpreted as an unconditioned overall association or a causal effect. Because GA at birth may lie on the pathway between PE and neonatal biomarker alterations, additional models including GA at birth in weeks as a continuous covariate were conducted as sensitivity analyses. These models retained all neonates, including both members of twin pairs, and used the same pregnancy-clustered sandwich standard errors as the primary neonatal models. Their estimates were interpreted as associations conditional on GA at birth and not as direct causal effects. The functional relationship between GA at birth and each natural-log-transformed neonatal biomarker was examined graphically. Multicollinearity between PE exposure and GA at birth was assessed using variance inflation factors (VIFs). The maximum VIF was 1.22, indicating no problematic multicollinearity in the gestational-age-adjusted models. Multicollinearity was not applicable to the primary biomarker models or exploratory Firth models because these models were univariable.

Analyses of associations between biomarkers and severe PE, RDS, neonatal thrombocytopenia, maternal platelet transfusion, maternal postpartum thrombocytopenia, and other neonatal morbidities were not part of the primary biomarker analysis and were considered post hoc and exploratory. Exploratory regression analyses were restricted to severe PE and RDS, and to neonatal thrombocytopenia, which was retained as a single explicitly declared rare-outcome analysis because of its relevance to the hematologic focus of the study.

All exploratory biomarker–outcome models were univariable and were estimated using Firth bias-reduced logistic regression [39,40]. The severe-PE analyses comprised five maternal biomarker models and were restricted to mothers with PE. The RDS analyses comprised five neonatal biomarker models, whereas the neonatal thrombocytopenia analyses comprised five maternal and five neonatal biomarker models; both neonatal outcome analyses included PE pregnancies (PE-exposed groups). For analyses of RDS and neonatal thrombocytopenia, only the first enrolled neonate from each pregnancy was retained according to the study identification order. Consequently, each pregnancy contributed a single neonatal observation, and no correction for within-pregnancy clustering was required in these Firth models.

Biomarker concentrations were log_2_-transformed before model fitting; therefore, the resulting odds ratios (ORs) represent the change in outcome odds associated with a twofold higher biomarker concentration. Profile penalized-likelihood 95% confidence intervals and penalized likelihood-ratio *p*-values were calculated. All models were reported irrespective of statistical significance, together with the active sample size and event count. The 20 biomarker–outcome associations were estimated using separate univariable Firth models. The Benjamini–Hochberg procedure was subsequently applied jointly to the 20 resulting penalized likelihood-ratio *p*-values to control the false discovery rate across the complete set of exploratory analyses. Firth regression was used to reduce small-sample and separation bias; however, it does not overcome the fundamental limitation imposed by the small event counts. Accordingly, these analyses were interpreted as strictly exploratory and hypothesis-generating.

Maternal platelet transfusion, maternal postpartum thrombocytopenia, and neonatal outcomes with fewer than 10 events other than neonatal thrombocytopenia were summarized descriptively using counts, percentages, and biomarker medians with interquartile ranges. No logistic regression models were fitted for these other rare outcomes. No multivariable predictive models, receiver operating characteristic (ROC) curves, data-derived cut-offs, pairwise area under the curve (AUC) comparisons, sensitivities, specificities, or predictive values were calculated.

A model-specific complete-case approach was used. Participants with an unavailable measurement were excluded only from models requiring that variable and remained eligible for all other applicable analyses. Missing values were not imputed, and the analyzable sample size was reported for each model.

## 3. Results

### 3.1. Baseline Characteristics

The propensity-score procedure initially matched 47 PE pregnancies to 47 control pregnancies. Immediately after matching, the mean |SMD| was 0.052 and the maximum |SMD| was 0.152, with no matching variable exceeding 0.20. Postmatching exclusions left 31 PE and 45 control pregnancies and disrupted the original 1:1 pair structure. Final-cohort SMDs showed some erosion of balance: eight of nine matching variables remained below 0.20, whereas aspirin use had |SMD| = 0.308. Complete baseline equivalence was therefore not assumed, and residual imbalance and differential attrition were considered during interpretation (Table S1).

The 31 women with PE and 45 control women delivered 34 and 47 neonates, respectively. Baseline characteristics are summarized in Table 1 and Table 2. Separately from the propensity-score balance diagnostics reported in Table S1, the SMDs presented in Table 1 and Table 2 were calculated as descriptive final-cohort between-group differences. PE-exposed neonates were born at a median GA of 33 weeks, compared with 36 weeks among controls. Although this difference did not reach conventional statistical significance (*p* = 0.07), the three-week difference was considered clinically important and was addressed in secondary gestational-age-at-birth-adjusted sensitivity analyses. The PE-exposed group also had a lower median five-minute Apgar score (8 vs. 9; *p* = 0.03) and a higher uterine artery mean pulsatility index (median 0.9 vs. 0.7; *p* = 0.01). The uterine artery finding was expected in the context of PE and reflects disease-related uteroplacental vascular pathology rather than a conventional baseline confounder. These *p*-values were interpreted descriptively.

Neonatal morbidity findings were considered descriptive because of the small number of events and the clinically important difference in GA between the groups. Neonatal thrombocytopenia was observed in 5 of 33 (15.2%) PE-exposed neonates and in 1 of 47 (2.1%) control neonates, whereas IVH occurred in 2 of 34 neonates in each group (5.9% and 4.3%, respectively). No regression analysis of PE exposure against these outcomes and no predictive-performance analysis were performed. IVH was not modeled inferentially; separate exploratory biomarker–thrombocytopenia Firth models within the PE-exposed group are confined to Table S3. They represent preliminary observations requiring confirmation in larger cohorts.

Maternal age was 34 ± 5.9 years in the PE group and 33 ± 5.7 years among controls, while median pre-pregnancy body mass index (BMI) was 26.3 and 23.3 kg/m^2^, respectively. Among women with PE, 19/31 (61.3%) had early-onset PE and 12/31 (38.7%) had late-onset PE. In total, 20 women (64.5%) had non-severe PE and 11 (35.5%) had severe PE. HELLP syndrome occurred in 2/31 women (6.5%), whereas no cases of PRES were recorded. Pharmacological induction using misoprostol and oxytocin was recorded in 27/31 (87.1%) PE pregnancies and 21/45 (46.7%) control pregnancies, whereas amniotomy was recorded in 4/31 (12.9%) and 24/45 (53.3%), respectively (*p* < 0.001). These labor-management findings are descriptive and likely reflect differences in clinical indication and GA. Persistent postpartum thrombocytopenia was observed in 3/31 (9.7%) women with PE and in none of the controls. Five women with PE received platelet transfusion (16.1%), whereas no control woman required platelet transfusion. These characteristics were not used to establish baseline equivalence or to select covariates according to statistical significance.

### 3.2. Primary Analyses

Descriptive summaries of maternal and neonatal laboratory measurements are presented in Table 3. The *p*-values are provided for descriptive transparency only. Maternal comparisons and neonatal categorical comparisons were unadjusted, whereas continuous neonatal laboratory comparisons were adjusted for GA at birth and the 5-min Apgar score using Quade’s rank ANCOVA, as indicated in the table footnote. Separately from the propensity-score balance diagnostics reported in Table S1, the SMDs presented in Table 3 were calculated as descriptive final-cohort between-group differences.

**Figure 3:**
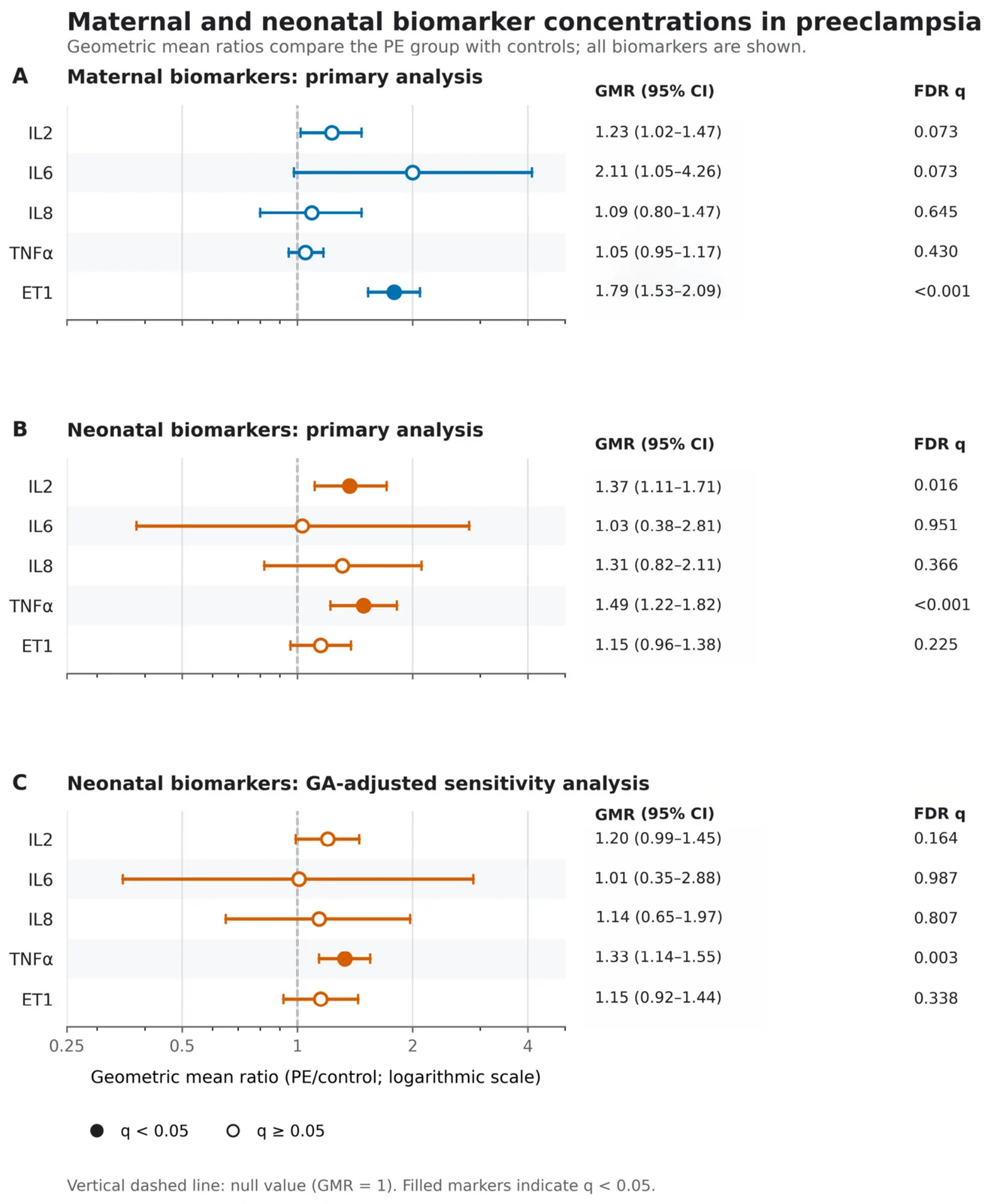
Primary comparisons of maternal and neonatal serum biomarker concentrations between the preeclampsia and control groups. Forest plots present geometric mean ratios (GMRs) with 95% confidence intervals (CIs) from linear models fitted to natural-log-transformed biomarker concentrations. (**A**) Maternal primary models using HC3 heteroscedasticity-consistent standard errors; (**B**) neonatal primary models using pregnancy-clustered standard errors to account for twins; (**C**) neonatal sensitivity models adjusted for gestational age using the same pregnancy-clustered approach. A GMR > 1 indicates a higher geometric mean biomarker concentration in the PE group. The vertical dashed line denotes the null value (GMR = 1). Filled markers indicate q < 0.05 and open markers q ≥ 0.05 after the corresponding Benjamini–Hochberg false discovery rate correction. The primary comparisons and gestational-age-adjusted sensitivity analyses were corrected as separate multiple-testing families. Two maternal IL6 concentrations (0.32 and 0.84 pg/mL) fell below the lowest non-zero calibration standard of 1 pg/mL and were therefore treated as non-quantifiable and excluded from all IL6-specific analyses. No fixed-value substitution or imputation was applied. Abbreviations: CI, confidence interval; ET1, endothelin 1; FDR, false discovery rate; GA, gestational age; GMR, geometric mean ratio; HC3, heteroscedasticity-consistent covariance estimator 3; IL, interleukin; PE, preeclampsia; TNFα, tumor necrosis factor α.

Inferential conclusions regarding the primary biomarkers were based on Table 4 and Figure 3. Among the 157 specimens, 124 IL2 and 57 IL6 concentrations were below 10 pg/mL, whereas no IL8 concentration was below 1 pg/mL and no TNFα concentration was below 2 pg/mL. Because the manufacturer reported the LLOQs as inequality bounds (<10, <10, <1, and <2 pg/mL, respectively), these counts describe concentrations below the numerical upper boundaries reported in the product insert and should not be interpreted as definitive counts below exact lot-specific LLOQs. The eight nominal calibration-standard concentrations spanned 1–2000 pg/mL for IL2, 1–3000 pg/mL for IL6, 1–2000 pg/mL for IL8, and 1–2000 pg/mL for TNFα. The archived curve-fit R^2^ values were 0.999991, 0.999939, 0.999998, and 0.999973, respectively (Table S2). All IL2 concentrations (range: 1.54–20.71 pg/mL) were within the study calibration range of 1–2000 pg/mL. Two maternal IL6 concentrations (0.32 and 0.84 pg/mL) fell below the lowest non-zero calibration standard of 1 pg/mL and were therefore treated as non-quantifiable and excluded from all IL6-specific analyses. No fixed-value substitution or imputation was applied.

Primary model-based between-group estimates, confidence intervals, and FDR-adjusted results are presented in Table 4 and Figure 3. After correction for the primary biomarker comparisons, maternal ET1 remained higher in the PE group (GMR = 1.79, 95% CI 1.53–2.09; q < 0.001). Maternal IL2 and IL6 showed nominal between-group differences, but neither remained statistically significant after FDR correction (GMR = 1.23, 95% CI 1.02–1.47, q = 0.073, and GMR = 2.11, 95% CI 1.05–4.26, q = 0.073, respectively). Regarding maternal IL6, two measurements were below the lowest non-zero calibration standard and were excluded. Neonatal IL2 (GMR = 1.37, 95% CI 1.11–1.71; q = 0.016) and TNFα (GMR = 1.49, 95% CI 1.22–1.82; q < 0.001) were also higher among PE-exposed neonates. In the GA-at-birth-adjusted sensitivity analysis, after applying the Benjamini–Hochberg correction across the five neonatal models, TNFα remained higher among PE-exposed neonates (GA-at-birth-adjusted GMR = 1.33, 95% CI 1.14–1.55; *p* < 0.001, q = 0.003). In contrast, the estimate for neonatal IL2 was attenuated and no longer met either the nominal or FDR-adjusted significance threshold (GA-at-birth-adjusted GMR = 1.20, 95% CI 0.99–1.45; *p* = 0.065, q = 0.164). No clear GA-conditional associations were observed for neonatal IL6, IL8, or ET1 (all q ≥ 0.338). No clear between-group differences were observed for the remaining maternal or neonatal biomarkers (Table 4, Figure 3).

The primary neonatal analyses included 81 neonates from 76 pregnancies. Both members of twin pairs were retained, and within-pregnancy dependence was accounted for using pregnancy-clustered sandwich standard errors.

### 3.3. Post Hoc Analyses

Post hoc exploratory regression analyses were restricted to severe PE, RDS, and neonatal thrombocytopenia at birth. One neonate was excluded because the PLT count at birth was unavailable. Post hoc exploratory Firth bias-reduced logistic regression analyses were conducted after retaining only the first enrolled neonate from each pregnancy. Consequently, each pregnancy contributed a single neonatal observation, and no correction for within-pregnancy clustering was required. Thus, the RDS analyses included 31 neonates, with 13 events, whereas the neonatal-thrombocytopenia analyses included 30 neonates, with 5 events. The separate severe-PE analyses included 31 PE mothers, of whom 11 had severe PE.

Post hoc exploratory Firth bias-reduced logistic regression analyses are presented in Table S3. None of the exploratory associations remained statistically significant after Benjamini–Hochberg correction (all q ≥ 0.120). Given the limited event counts, these analyses were considered strictly hypothesis-generating. Because none of these associations remained statistically significant after FDR correction and several confidence intervals were markedly wide, particularly that for neonatal ET1, these results should not be interpreted as evidence of independent or predictive effects. Accordingly, no independent biomarker association, predictive performance, or clinically applicable risk-stratification inference can be supported by these analyses.

Neonatal thrombocytopenia at birth occurred in 5/33 (15.2%) PE-exposed neonates and 1/47 (2.1%) control neonates. These frequencies and the corresponding outcome-positive biomarker distributions are presented descriptively in Table 1 and Table S4.

Other rare maternal and neonatal outcomes are additionally summarized descriptively in Table S4. All five maternal transfusions occurred among mothers with PE and consisted exclusively of platelet transfusions (5/31, 16.1%; controls: 0/45). Postpartum maternal thrombocytopenia was recorded in 3/31 (9.7%) mothers with PE and in none of the controls. Among the 12 neonates born at <32 GW in each group, one neonate per group died from late-onset sepsis before reaching 36^+0^ weeks’ PMA and was therefore unevaluable for BPD. All remaining eligible neonates underwent assessment at 36^+0^ weeks’ PMA. BPD was identified in 5/11 (45.5%) PE-exposed neonates and 2/11 (18.2%) controls. All 34 PE-exposed and 47 control neonates underwent cranial ultrasonography within the first 24 h of life. Thirteen PE-exposed and 15 control neonates met the gestational-age or birthweight criteria for routine serial examinations on postnatal days 1, 3, and 7. IVH was identified in 2/34 (5.9%) PE-exposed neonates and 2/47 (4.3%) controls. The two PE-exposed cases were grade II and grade III, whereas both control cases were grade II. Because the intensity of subsequent surveillance differed according to GA, birthweight, initial findings, and clinical indications, IVH was retained as a descriptive outcome without inferential biomarker modeling. NEC occurred in 2/34 (5.9%) PE-exposed neonates and 1/47 (2.1%) controls; among neonates eligible for ROP screening, ROP was identified in 3/13 (23.1%) and 1/15 (6.7%), respectively.

Table S4 also presents the median (IQR) concentrations of all five relevant maternal or neonatal biomarkers among outcome-positive participants with complete biomarker measurements. Depending on the outcome, these summaries were based on three to seven active participants and were therefore considered descriptive only. No inferential comparisons are presented in Table S4; the separate exploratory Firth analyses of neonatal thrombocytopenia are reported in Table S3.

## 4. Discussion

### 4.1. Main Findings

This secondary report from a single-center observational cohort primarily aimed to characterize maternal and early neonatal serum cytokine and ET1 levels associated with PE. In the primary analyses, maternal ET1 concentrations were higher in the PE group and remained significant after FDR correction (GMR = 1.79, 95% CI: 1.53–2.09; q < 0.001). Maternal IL2 and IL6 showed nominal between-group differences, but neither remained statistically significant after FDR correction (q = 0.073 for both).

Among neonates, IL2 and TNFα concentrations were higher in the PE-exposed group in the primary pregnancy-clustered analyses and remained significant after FDR correction. In the GA-at-birth-adjusted sensitivity analyses, the association with neonatal TNFα persisted (GMR = 1.33, 95% CI: 1.14–1.55; q = 0.003), whereas the estimate for neonatal IL2 was attenuated and no longer met the significance threshold. No clear GA-conditional associations were observed for neonatal IL6, IL8, or ET1.

The post hoc Firth analyses were restricted to severe PE, RDS, and neonatal thrombocytopenia among PE-exposed mothers and neonates. None of the exploratory biomarker–outcome associations remained statistically significant after FDR correction. Given the small event counts and wide confidence intervals, these analyses were considered strictly hypothesis-generating.

The descriptive findings provide additional clinical context. Neonatal thrombocytopenia at birth occurred in 5/33 PE-exposed and 1/47 control neonates, whereas IVH occurred in 2/34 and 2/47 neonates, respectively. BPD, NEC, and ROP were also uncommon, and their biomarker distributions were summarized descriptively without inferential comparisons. These observations do not support biomarker-based prediction, clinically applicable thresholds, or causal mechanisms linking the investigated biomarkers to neonatal complications.

### 4.2. Interpretation

Maternal ET1 represented the clearest maternal biomarker difference, remaining higher in the PE group after FDR correction. This result is compatible with the endothelial dysfunction characteristic of PE, but the cross-sectional design does not establish that ET1 reflects disease severity, the degree of placental ischemia, or a specific causal pathway. A previous serum study also evaluated ET1 in PE and HELLP syndrome, although the PE–control difference was not statistically significant and the largest elevations were reported in HELLP syndrome [35]. Maternal IL2 and IL6 showed nominal between-group differences, but neither remained statistically significant after FDR correction and neither should therefore be interpreted as a confirmed maternal biomarker difference. The wider literature is likewise heterogeneous: one study reported higher maternal IL6 and TNFα concentrations in PE, whereas another found no difference in maternal or cord-serum IL6 [34,41].

Neonatal IL2 and TNFα concentrations were higher among PE-exposed neonates in the primary pregnancy-clustered analyses. However, these findings do not establish an inflammatory alteration independent of GA or other perinatal factors. Available cord-blood studies provide some support for the TNFα finding [42,43], although their biological compartment and sampling time differ from the radial-artery samples obtained during the first postnatal hour in the present study. In the GA-at-birth-adjusted sensitivity analysis, the TNFα difference persisted, whereas the IL2 estimate was attenuated and no longer met either the nominal or FDR-adjusted significance threshold. Neonatal TNFα is therefore the more robust neonatal signal in this cohort, while the IL2 finding may partly reflect GA at birth or other unmeasured perinatal factors.

Delivery management differed between the study groups, with pharmacological induction being more frequent among PE pregnancies and amniotomy more frequent among controls. These differences should not be interpreted as causal determinants of neonatal outcomes because the timing and method of delivery were selected according to clinical indications and were closely related to PE severity, fetal condition, and GA. Previous evidence also emphasizes the importance of GA and fetal condition when interpreting induction outcomes [44]; however, the present study was not designed to compare delivery strategies.

Neonatal thrombocytopenia at birth was more frequent descriptively in the PE-exposed group (5/33) than in controls (1/47). None of the exploratory biomarker–thrombocytopenia associations remained statistically significant after FDR correction, and all estimates were based on only five events. The especially wide confidence interval for neonatal ET1 further indicates limited precision. Neonatal thrombocytopenia is recognized among neonates exposed to PE or gestational hypertension, particularly among preterm infants [45,46,47]. Placental insufficiency and fetal hypoxia have been proposed as potential contributors through impaired fetal megakaryopoiesis and thrombopoiesis [2,48,49,50]. Previous studies have emphasized prematurity and placental insufficiency rather than cytokine or ET1 associations [45,51]. However, because placental histology, cord-blood gases, lactate concentrations, and fetal PLT production were not assessed, this pathway provides biological context only and was not demonstrated by the present cohort. BPD, IVH, NEC, and ROP were similarly represented by very few events and were therefore reported descriptively without biomarker–outcome inference. Their observed biomarker distributions do not support biomarker-specific associations, between-group risk attribution, vascular-remodeling claims, or mechanistic conclusions.

The broader neonatal literature supports the general biological relevance of inflammatory and endothelial pathways but does not validate outcome-specific associations in the present cohort. BPD has been linked to inflammatory and vascular-development pathways [52,53,54,55], with human studies examining IL6, TNFα, and IL8 [56], experimental models supporting biological involvement of IL6 and TNFα [57,58], and ET1 also evaluated as a candidate biomarker [59]; however, the broader literature remains heterogeneous [60]. Other studies have evaluated inflammatory or endothelial biomarkers in relation to IVH [61,62], ROP [63], NEC [64], and RDS [36,65,66,67]. These investigations involved different populations, biological compartments, sampling times, outcome definitions, and analytical designs; consequently, they cannot convert the sparse descriptive findings of the present study into clinically applicable associations.

In addition to inflammatory and endothelial biomarkers, markers of coagulation activation may provide complementary information in complicated pregnancies. Fibrin monomer (FM), commonly assessed as soluble fibrin monomer complex (SFMC), reflects ongoing fibrin formation and has been proposed as a potential marker of thrombotic activation in pregnancy. In a recent observational study of 100 third-trimester pregnancies, SFMC and D-dimer concentrations did not differ significantly between women classified as having low or high venous-thromboembolism risk according to the Royal College of Obstetricians and Gynaecologists score, emphasizing that the clinical value of this marker remains uncertain [68]. FM was not measured in the present study; therefore, no relationship between FM, the investigated inflammatory and endothelial biomarkers, or PE-related maternal and neonatal outcomes can be inferred. Future studies combining inflammatory, endothelial, and hemostatic markers may help to characterize these overlapping biological pathways more comprehensively.

Taken together, maternal ET1 and early neonatal TNFα were the most robust between-group findings, whereas the neonatal IL2 difference was attenuated after adjustment for GA at birth. None of the post-hoc complication-related associations remained statistically significant after FDR correction. These findings do not support predictive or causal inference and require replication in larger prospective cohorts.

### 4.3. Strengths and Limitations

The principal limitations of this study are its single-center observational design, relatively small cohort, and limited number of maternal and neonatal outcome events. These characteristics restrict generalizability, preclude causal inference, and leave the findings susceptible to residual confounding. The small event counts did not support reliable multivariable modeling or evaluation of predictive performance. Although Firth regression was used to reduce small-sample and separation bias in the selected post hoc analyses, it cannot compensate for the limited information provided by sparse outcomes. The wide confidence intervals and the absence of associations remaining significant after FDR correction emphasize the uncertainty of these exploratory estimates.

The original 1:1 matched structure was disrupted by asymmetric postmatching exclusions, principally because declined or withdrawn consent was more frequent among PE pregnancies. Retaining eligible, consenting controls preserved validly collected observations but converted the final cohort into an unpaired selected sample. Because antenatal corticosteroid exposure could occur downstream of PE diagnosis, its inclusion in the matching model may have attenuated part of the PE-associated biomarker contrast; consequently, the findings should not be interpreted as estimating the unconditioned total effect of PE. Final-cohort SMDs demonstrated some erosion of balance, including residual imbalance in aspirin use, and differential attrition may have introduced selection bias. Accordingly, residual confounding cannot be excluded and the primary estimates should not be generalized as causal ATT effects.

GA at birth differed by approximately three weeks between the groups and is strongly related to several neonatal outcomes and biomarker concentrations. GA at recruitment, which was used in the matching procedure, was recorded at the initial eligibility assessment and was distinct from GA at diagnosis or delivery. Because GA at birth may lie partly on the pathway between PE and neonatal alterations, it cannot be treated solely as a conventional confounder. The primary neonatal models therefore estimated the between-group contrast in the final design-selected cohort without additional adjustment for GA at birth, whereas the gestational-age-at-birth-adjusted models were treated as sensitivity analyses estimating conditional associations. Neither analysis can fully separate the effects of PE, prematurity, postmatching selection, and other unmeasured perinatal factors.

The inclusion of both singleton and twin pregnancies introduced additional clinical heterogeneity because plurality may influence GA at delivery, fetal growth, delivery management, and neonatal outcomes. Pregnancy-clustered standard errors accounted for the statistical non-independence of neonates from the same pregnancy but could not eliminate differences in underlying clinical risk between singleton and twin pregnancies. Only three twin pregnancies were included in the PE group and two in the control group; therefore, reliable plurality-stratified analyses were not feasible. The neonatal findings should consequently be interpreted as estimates from a mixed singleton–twin cohort.

Additional limitations relate to sampling and biomarker measurement. Maternal and neonatal biomarkers were measured at a single time point, preventing assessment of their temporal trajectories. The timing of maternal sampling relative to magnesium sulfate, antihypertensive treatment, and antenatal corticosteroids was not protocol-standardized, and neonatal sampling relative to resuscitation, oxygen administration, and respiratory support depended on routine clinical care. The manufacturer reported the AimPlex LOD and LLOQ as inequality bounds rather than exact lot-specific numerical thresholds. Although all IL2 concentrations were within the study calibration range and no fixed-value substitution was applied, most IL2 concentrations were below 10 pg/mL; therefore, the quantitative precision of measurements toward the lower end of the calibration curve should be considered when interpreting the IL2 findings. In addition, because specimens were measured in single determinations, independent within-study replicate coefficients of variation could not be estimated. The consistent laboratory setting, instrument, personnel, analytical protocol, and common calibration function reduced procedural variability, although residual between-cycle variation cannot be completely excluded. A model-specific complete-case approach was used without imputation. Furthermore, placental histology, cord-blood gases, lactate concentrations, fetal PLT production, and other direct measures of placental ischemia or fetal hypoxia were not assessed. Consequently, the study cannot establish the biological mechanisms linking PE, biomarker concentrations, and neonatal outcomes.

Our study likewise shares the main inherent limitations of cytokine research. Cytokine concentrations are sensitive to sampling time, biological compartment, specimen handling, and assay methodology, while their rapid kinetics and short half-lives can complicate the interpretation of single measurements [69,70]. Reported diagnostic performance and proposed thresholds vary across studies, and no single cytokine can be regarded as a diagnostic gold standard [14,15]. Consequently, although several cytokines are consistently elevated in specific neonatal diseases, single-cytokine testing is not sufficiently reliable for prediction or diagnosis. Combining cytokines with established inflammatory markers, such as C-reactive protein, may provide greater diagnostic accuracy than individual cytokines, although this was not evaluated in the present study [14,15].

The study also has several strengths. Its primary objective—the comparison of maternal and neonatal cytokine and ET1 concentrations between PE and control groups—was defined within the original study framework. Concurrent maternal and neonatal sampling enabled characterization of both components of the between-group biomarker differences associated with PE at delivery. Maternal blood was collected before placental delivery, during labor in women who delivered vaginally, and before transfer to the operating room in women who underwent cesarean delivery. Neonatal blood was obtained from the radial artery within the first hour of life, rather than from cord blood. The use of early peripheral neonatal blood rather than cord blood provided information on the circulating neonatal biomarker concentrations during the immediate postnatal period. Samples were processed under standardized conditions, stored for no longer than six months, subjected to a single freeze–thaw cycle, and analyzed in batches by personnel blinded to group allocation and clinical outcomes.

Strict eligibility criteria reduced several major sources of inflammatory heterogeneity, including diabetes mellitus, autoimmune disease, antiphospholipid syndrome, recent COVID-19, and clinical chorioamnionitis. All five maternal and five neonatal biomarker results were reported irrespective of statistical significance, effect estimates were accompanied by 95% confidence intervals, and FDR correction was applied to the specified multiple-testing families. The primary neonatal analyses retained all available neonates while accounting for the non-independence of twins through pregnancy-clustered standard errors. Together, these design and analytical features strengthen the internal consistency and transparency of the primary findings, while the post hoc outcome analyses remain explicitly hypothesis-generating.

## 5. Conclusions

In this single-center observational cohort, PE was associated with higher maternal ET1 and early neonatal TNFα concentrations. The between-group difference in neonatal IL2 was attenuated after adjustment for GA at birth. The complication-related analyses were post hoc, were limited by sparse event counts, and yielded no associations that remained statistically significant after FDR correction; consequently, neither predictive nor causal inference can be supported. Replication of the primary maternal and neonatal biomarker findings in larger prospective cohorts is required.

## Figures and Tables

**Table 1:** Perinatal and clinical characteristics and neonatal outcomes of neonates born to women with preeclampsia and pregnant controls. Continuous variables are presented as the mean ± standard deviation (SD) or median (interquartile range [IQR]), as appropriate, and categorical variables as n/N (%). *p*-values are descriptive and were not used to establish group comparability or to select adjustment covariates. Neonates constitute the unit of description; the non-independence of twins was addressed in the neonatal biomarker models and sensitivity analyses. All *p*-values in this table should be interpreted descriptively. The SMDs for neonatal variables quantify descriptive post-exposure differences and were not used to assess the adequacy of maternal propensity-score matching.

Neonates	Neonates Born to PE ^a^ Mothers (N = 34)	Neonates Born to Healthy Mothers (N = 47)	*p*-Value	|SMD| ^o^
GA ^b^ at birth (weeks)	33 (30–36)	36 (31.5–38)	0.07 *	0.373
Gender (male)	17/34 (50%)	26/47 (55.3%)	0.64	0.107
Term	6/34 (17.6%)	15/47 (31.9%)	0.15	0.335
Preterm	28/34 (82.4%)	32/47 (68.1%)	0.15	0.335
Extremely preterm	4/28 (14.2%)	4/32 (12.5%)	1.00 **	0.052
Very preterm	8/28 (28.6%)	8/32 (25%)	0.76	0.081
Moderate preterm	8/28 (28.6%)	8/32 (25%)	0.76	0.081
Late preterm	8/28 (28.6%)	12/32 (37.5%)	0.46	0.191
Birthweight (grams)	2035 (1475–2752.5)	2450 (1435–3140)	0.27 *	0.243
Below 1000 g	7/34 (20.6%)	5/47 (10.6%)	0.21	0.277
Below 2500 g	22/34 (64.7%)	25/47 (53.2%)	0.30	0.236
Birthweight percentile	46.8 ± 30.7	45.3 ± 28	0.82	0.051
Length (cm)	44.4 (36–49)	47 (40.5–50)	0.20 *	0.301
Length percentile	57 (26.8–86.3)	56 (24.8–86.4)	0.79 *	0.008
Head circumference (cm)	31 (28.3–33.4)	32.5 (29.2–34.5)	0.39 *	0.006
Circumference percentile	54.5 (42.8–87.3)	68.9 (42.5–83.5)	0.86 *	0.034
SGA ^c^	4/34 (11.8%)	5/47 (10.6%)	1.00 **	0.036
IUGR ^d^	8/34 (23.5%)	10/47 (21.3%)	0.81	0.054
**Pregnancy characteristics**				
Twins	6/34 (17.6%)	4/47 (8.5%)	0.31 **	0.273
ART ^e^	12/34 (35.3%)	13/47 (27.7%)	0.46	0.165
Umbilical artery PI ^f^	1 ± 0.2	1 ± 0.1	0.77	0.103
**Delivery characteristics**				
Delivery type (CS ^g^)	33/34 (97.1%)	42/47 (89.4%)	0.39 **	0.310
Placenta weight (grams)	385 (302–487.5)	450 (390–500)	0.10 *	0.157
Apgar score at 1 min	8 (7–8)	8 (7–8)	0.50 *	0.103
Apgar score at 5 min	8 (8–9)	9 (8–9)	0.03 *	0.425
**Neonatal major complications**				
NICU ^h^ admission	24/34 (70.6%)	24/47 (51.1%)	0.08	0.408
Respiratory assistance	21/34 (61.8%)	26/47 (55.3%)	0.56	0.131
Infection	11/34 (32.4%)	9/47 (19.2%)	0.17	0.305
Sepsis	4/34 (11.8%)	2/47 (4.3%)	0.23 **	0.279
RDS ^i^	13/34 (38.2%)	14/47 (29.8%)	0.43	0.179
BPD ^j^, assessed at 36 weeks PMA ^k^	5/11 (45.5%)	2/11 (18.2%)	0.36 **	0.612
IVH ^l^, any grade	2/34 (5.9%)	2/47 (4.3%)	1.00 **	0.074
NEC ^m^, modified Bell stage ≥ II	2/34 (5.9%)	1/47 (2.1%)	0.57 **	0.192
ROP ^n^, any stage	3/13 (23.1%)	1/15 (6.7%)	0.31 **	0.474
Thrombocytopenia (<150 × 10^9^/L)	5/33 (15.2%)	1/47 (2.1%)	0.08 **	0.477
Any neonatal transfusion	7/34 (20.6%)	11/47 (23.4%)	0.76	0.068
Death	1/34 (2.9%)	1/47 (2.1%)	1.00 **	0.052

^a^ PE: preeclampsia; ^b^ GA: gestational age at birth; ^c^ SGA: small for gestational age; ^d^ IUGR: intrauterine growth restriction; ^e^ ART: assisted reproductive technology; ^f^ PI: pulsatility index; ^g^ CS: cesarean section; ^h^ NICU: neonatal intensive care unit; ^i^ RDS: respiratory distress syndrome; ^j^ BPD: bronchopulmonary dysplasia; ^k^ PMA: postmenstrual age; ^l^ IVH: intraventricular hemorrhage; ^m^ NEC: necrotizing enterocolitis; ^n^ ROP: retinopathy of prematurity; ^o^ SMD: standardized mean difference. The BPD denominator included neonates born at <32 GW who survived to and were assessed at 36^+0^ weeks’ PMA. One eligible neonate in each group died from late-onset sepsis before reaching the assessment time point and was therefore excluded from the denominator. ROP percentages were calculated among neonates eligible for screening and examined according to the study protocol (13 PE-exposed and 15 control neonates, respectively, born at <32 GW or with a birthweight <1500 g). An early PLT count was unavailable for 1 PE-exposed neonate; therefore, the denominator for neonatal thrombocytopenia was 33 in the PE group. All *p*-values in this table should be interpreted descriptively. Regarding numerical variables, *p*-values were calculated using the two-sample *t*-test when the numerical variable followed a normal distribution across the two groups. Regarding categorical variables, *p*-values were calculated using the chi-square test. * *p*-values with one asterisk indicate that the Mann–Whitney U test was conducted, since the numerical variable did not follow the normal distribution across the groups. ** *p*-values with two asterisks (**) indicate that Fisher’s exact test was conducted, utilized when the expected numbers were very small (expected frequencies less than 1, or more than 20% of the expected frequencies less than 5). The reported |SMD| values are descriptive final-cohort standardized differences calculated using pooled group variances. They are not propensity-score matching diagnostics. The descriptive *p*-values and SMDs in this table were not adjusted for within-pregnancy clustering and should not be interpreted inferentially.

**Table 2:** Maternal demographic, obstetric, and clinical characteristics of the preeclampsia and control groups. Continuous variables are presented as the mean ± standard deviation (SD) or median (interquartile range [IQR]), as appropriate, and categorical variables as n/N (%). Women constitute the unit of description. All comparisons are descriptive; no hypothesis testing was used to establish group equivalence or to select adjustment covariates. All *p*-values in this table should be interpreted descriptively. The SMD column describes the final analytic cohort after postmatching exclusions; balance was also assessed before and immediately after matching as described in Section 2.4.

Pregnant Women	PE ^a^ Group (N = 31)	Pregnant Controls (N = 45)	*p*-Value	|SMD| ^h^
Age (years)	34 ± 5.9	33 ± 5.7	0.45	0.172
Maternal age ≥ 40 years	4/31 (12.9%)	5/45 (11.1%)	1.00 **	0.055
Maternal age < 18 years	1/31 (3.2%)	0/45 (0%)	0.41 **	0.258
Gestational age at recruitment (weeks)	29.0 (25.5–31.0)	28.0 (25.0–30.0)	0.16 *	0.101
Race	All Caucasian	All Caucasian	-	-
BMI ^b^ before (kg/m^2^)	26.3 (23.5–28.4)	23.3 (21.2–27.7)	0.06 *	0.145
BMI at labor (kg/m^2^)	29.6 (28.8–31.2)	28.3 (25.3–31.6)	0.12 *	0.351
BMI difference (kg/m^2^)	3.6 (1.7–5)	3.4 (2.5–5.5)	0.57 *	0.134
Smoking before pregnancy	7/31 (22.6%)	15/45 (33.3%)	0.31	0.241
Smoking during pregnancy	2/31 (6.5%)	9/45 (20%)	0.18 **	0.408
**Pregnancy characteristics**				
Mean uterine artery’s PI ^c^	0.9 (0.8–1.5)	0.7 (0.6–0.9)	0.01 *	0.993
**Obstetric history**				
Gravidity	2 (1–3)	2 (2–3)	0.06 *	0.465
Parity	1 (1–2)	2 (1–2)	0.049 *	0.412
PE in previous pregnancies	3/31 (9.7%)	3/45 (6.7%)	0.68 **	0.110
Pregnancy losses	0 (0–0.5)	1 (0–1)	0.03 *	0.435
**Medication exposures documented before biospecimen collection**				
Aspirin	21/31 (67.7%)	24/45 (53.3%)	0.21	0.298
LMWH ^d^	12/31 (38.7%)	16/45 (35.6%)	0.78	0.065
Progesterone	6/31 (19.4%)	11/45 (24.4%)	0.60	0.123
Thyroxine	14/31 (45.2%)	17/45 (37.8%)	0.52	0.150
Steroids	21/31 (67.7%)	28/45 (62.2%)	0.62	0.116
**Comorbidities and major complications**				
Hypothyroidism	14/31 (45.2%)	17/45 (37.8%)	0.52	0.150
Hypercholesterolemia	1/31 (3.2%)	4/45 (8.9%)	0.64 **	0.239
Persistent thrombocytopenia after delivery	3/31 (9.7%)	0/45 (0%)	0.06 **	0.463
Platelet transfusion	5/31 (16.1%)	0/45 (0%)	0.009 **	0.620
ICU ^e^ admission	1/31 (3.2%)	0/45 (0%)	0.41 **	0.258
Death	0/31 (0%)	0/45 (0%)	-	-
**Labor management**				
Pharmacological induction	27/31 (87.1%)	21/45 (46.7%)	<0.001	0.951
Amniotomy	4/31 (12.9%)	24/45 (53.3%)	<0.001	0.951
**PE type**				
Early-onset PE	19/31 (61.3%)	Not applicable	-	-
Late-onset PE	12/31 (38.7%)	Not applicable	-	-
Severe PE	11/31 (35.5%)	Not applicable	-	-
Non-severe PE	20/31 (64.5%)	Not applicable	-	-
HELLP ^f^ syndrome	2/31 (6.5%)	Not applicable	-	-
PRES ^g^	0/31 (0%)	Not applicable	-	-

^a^ PE, preeclampsia; ^b^ BMI, body mass index; ^c^ PI, pulsatility index; ^d^ LMWH, low-molecular-weight heparin; ^e^ ICU, intensive care unit; ^f^ HELLP, hemolysis, elevated liver enzymes, low platelet syndrome; ^g^ PRES, posterior reversible encephalopathy syndrome; ^h^ SMD: standardized mean difference. All *p*-values in this table should be interpreted descriptively. Regarding numerical variables, *p*-values were calculated using the two-sample *t*-test when the numerical variable followed a normal distribution across the two groups. Regarding categorical variables, *p*-values were calculated using the chi-square test. * *p*-values with one asterisk (*) indicate that the Mann–Whitney U test was conducted, since the numerical variable did not follow the normal distribution across the groups. ** *p*-values with two asterisks (**) indicate that Fisher’s exact test was conducted, utilized when the expected numbers were very small (expected frequencies less than 1, or more than 20% of the expected frequencies less than 5). Maternal age < 18 years and ≥40 years was reported descriptively to describe minor-age pregnancy and very advanced maternal age, respectively. One participant met the ≥18-year eligibility criterion according to the identification documentation available at recruitment; subsequent official verification during subsequent verification of the study records after enrollment established that she was 17 years old. The corrected age is reported in the table. Early-/late-onset PE and non-severe/severe PE represent separate classification dimensions. The *p*-value for labor management compares the distribution of the two categories using Pearson’s chi-squared test. The |SMD| values in this table are descriptive final-cohort SMDs calculated using pooled group variances. Consequently, for covariates included in the propensity-score model, they differ slightly from the final-cohort SMDs reported in Table S1, which were standardized using the corresponding pre-matching PE-group standard deviation under the ATT estimand. The two approaches produced very similar values and did not materially change the assessment of residual imbalance.

**Table 3:** Maternal and neonatal results in the preeclampsia and control groups. Continuous variables are presented as mean ± standard deviation (SD) or median (interquartile range [IQR]), as appropriate; categorical variables are presented as n/N (%). All *p*-values in this table should be interpreted descriptively.

	PE ^a^ Group (N = 31)	Pregnant Controls (N = 45)	*p*-Value	|SMD| ^p^	Neonates Born to PE Mothers (N = 34)	Neonates Born to Healthy Mothers (N = 47)	*p*-Value	|SMD| ^p^
Platelet count × 10^9^/L	167 (138.5–201.5)	234 (197–271)	<0.001 *	1.031	230 (168–257)	282 (226.5–322.5)	0.002 ^^^	1.040
MPV ^b^ (fL)	9.8 (8.4–10.8)	10.2 (9.4–10.7)	0.61 *	0.016	7.4 (6.9–8)	9.1 (7.4–9.8)	0.01 ^^^	0.726
Platelet count <100 × 10^9^/L	3/31 (9.7%)	0/45 (0%)	0.06 **	0.463	0/33 (0%)	0/47 (0%)	-	0.000
Platelet count <150 × 10^9^/L	12/31 (38.7%)	1/45 (2.2%)	<0.001	1.014	5/33 (15.2%)	1/47 (2.1%)	0.08 **	0.477
RBCs ^c^ (×10^6^/μL)	3.9 ± 0.4	3.9 ± 0.4	0.93	0.022	4.7 ± 0.7	4.6 ± 0.7	0.75 ^^^	0.084
Hematocrit (%)	35 ± 3.9	34.8 ± 3.1	0.80	0.065	52.1 ± 6.5	49.3 ± 7.9	0.13 ^^^	0.392
Hemoglobin (g/dL)	11.8 ± 1.3	11.7 ± 1	0.66	0.114	17.4 (16–18.8)	17.1 (15.1–18)	0.25 ^^^	0.303
WBCs ^d^ (×10^3^/μL)	11.8 (9.6–13.6)	10.8 (8.7–12.6)	0.32 *	0.127	9.6 (6.5–14.7)	12.8 (10.2–15.8)	0.02 ^^^	0.642
Neutrophils (%)	77.6 ± 6.7	74.3 ± 7.3	0.07	0.476	39 ± 16.3	47.7 ± 14.3	0.03 ^^^	0.569
Lymphocytes (%)	15 ± 5.8	17.1 ± 6.2	0.19	0.337	47.8 ± 17.3	36.2 ± 14.8	0.007 ^^^	0.717
Creatinine (mg/dL)	0.72 ± 0.12	0.55 ± 0.08	<0.001 ***	1.667	0.69 ± 0.13	0.6 ± 0.13	0.04 ^^^	0.648
Urea (mg/dL)	22 (17–27)	14 (12–16)	<0.001 *	1.344	31.4 ± 14.7	19.5 ± 8.3	0.004 ^^^	0.994
AST ^e^ (U/L)	37 (32.5–98.5)	17 (15–21)	<0.001 *	1.092	42 (36–57)	45 (38–54)	1.00 ^^^	0.234
ALT ^f^ (U/L)	56 (46.5–148.5)	12 (8–18)	<0.001 *	1.234	7 (5–9.8)	10 (6–12)	0.08 ^^^	0.160
gGT ^g^ (U/L)	20 (12.5–25)	-	-	-	111 (60.3–182.5)	113 (85.3–201.3)	0.81 ^^^	0.103
ALP ^h^ (U/L)	130 (93–152)	123.5 (97.3–156.3)	0.94 *	0.075	189.4 ± 60.7	181.2 ± 51.3	0.67 ^^^	0.145
CRP ^i^ (mg/dL)	0.8 (0.38–1.21)	0.47 (0.14–1.05)	0.26 *	0.138	0.18 (0.07–0.42)	0.12 (0.04–0.43)	0.43 ^^^	0.211
LDH ^j^ (U/L)	257 (241–316)	201 (185–213)	<0.001 *	1.276	-	-	-	-
24-h urine protein (mg/24h)	551.8 (475–2627.1)	-	-	—	-	-	-	-
IL2 ^k^ (pg/mL)	4.4 (3.4–4.9)	3.2 (2.6–4.5)	0.02 *	0.499	5 (4.4–5.8)	4.5 (2.6–5.4)	0.008 ^^^	0.616
IL6 ^l^ (pg/mL)	6.9 (3.2–18.8) N = 30 ^§^	2.5 (1.3–5.9) *N* = 44 ^§^	0.006 *	0.078	69.8 (27–170.4)	86.1 (11–326.8)	0.99 ^^^	0.036
IL8 ^m^ (pg/mL)	3.5 (3.1–4.6)	3.4 (2.4–4.4)	0.39 *	0.091	9.5 (4.8–28.1)	6.3 (4.6–13.9)	0.32 ^^^	0.288
TNFα ^n^ (pg/mL)	9.8 ± 1.8	9.4 ± 2.2	0.42	0.206	13.4 (10.5–14.9)	10.1 (8.5–11.6)	<0.001 ^^^	0.471
ET1 ^o^ (pg/mL)	1 (0.9–1.4)	0.6 (0.5–0.6)	<0.001 *	1.760	1.2 (1–1.6)	1.2 (0.9–1.4)	0.27 ^^^	0.366
Antithrombin III (%)	92.8 ± 14.8	97.6 ± 9.9	0.13 ***	0.389	-	-	-	-
Protein C activity (%)	101 (86.5–115.5)	103 (97–111)	0.29 *	0.225	-	-	-	-
Free Protein S Antigen (%)	28.7 ± 4.9	39.5 ± 6.3	<0.001	1.902	-	-	-	-

^a^ PE, preeclampsia; ^b^ MPV, mean platelet volume; ^c^ RBCs, red blood cells; ^d^ WBCs, white blood cells; ^e^ AST, aspartate transaminase; ^f^ ALT, alanine transaminase; ^g^ gGT, gamma-glutamyltransferase; ^h^ ALP, alkaline phosphatase; ^i^ CRP, C-reactive protein; ^j^ LDH, lactate dehydrogenase; ^k^ IL2, interleukin 2; ^l^ IL6, interleukin 6; ^m^ IL8, interleukin 8; ^n^ TNFα, tumor necrosis factor α; ^o^ ET1, endothelin 1; ^p^ SMD: standardized mean difference. Maternal *p*-values and neonatal categorical *p*-values are unadjusted. *p*-values marked with a caret (^^^) were adjusted for gestational age at birth and the 5-min Apgar score using Quade’s rank ANCOVA. Regarding numerical variables, *p*-values were calculated using the two-sample *t*-test when the numerical variable followed the normal distribution across the two groups. Regarding categorical variables, *p*-values were calculated using the chi-square test. * *p*-values with one asterisk (*) indicate that the Mann–Whitney U test was conducted, since the numerical variable did not follow the normal distribution across the groups. ** *p*-values with two asterisks (**) indicate that Fisher’s exact test was conducted, utilized when the expected numbers were very small (expected frequencies less than 1, or more than 20% of the expected frequencies less than 5). *** *p*-values with three asterisks (***) indicate that Welch’s test was conducted, utilized when the assumption of homogeneity of variances for the two groups was not met. The reported |SMD| values are descriptive final-cohort standardized differences calculated using pooled group variances. They are not propensity-score matching diagnostics. ^§^ Two maternal IL6 concentrations (0.32 and 0.84 pg/mL) fell below the lowest non-zero calibration standard of 1 pg/mL and were therefore treated as non-quantifiable and excluded from all IL6-specific analyses. No fixed-value substitution or imputation was applied.

**Table 4:** Primary comparisons of maternal and neonatal serum biomarker concentrations between the preeclampsia and control groups.

Sample	Biomarker	PE ^a^ Group Median (IQR ^b^)	N	Control Group Median (IQR)	N	Primary Analysis			GA ^f^-Adjusted Sensitivity Analysis		
						GMR ^c^ (95% CI ^d^)	*p*-Value	FDR ^e^ q-Value	GMR (95% CI)	*p*-Value	FDR ^e^ q-Value
**Maternal**	IL2 ^g^	4.39 (3.39–4.88)	31	3.22 (2.64–4.50)	45	1.23 (1.02–1.47)	0.029	0.073	—	—	—
	IL6 ^h^	6.99 (3.17–18.81)	30 *^§^*	2.51 (1.29–5.94)	44 *^§^*	2.11 (1.05–4.26)	0.036	0.073	—	—	—
	IL8 ^i^	3.47 (3.15–4.63)	31	3.41 (2.35–4.41)	45	1.09 (0.80–1.47)	0.580	0.645	—	—	—
	TNFα ^j^	9.64 (8.75–10.63)	31	9.23 (7.72–10.25)	45	1.05 (0.95–1.17)	0.344	0.430	—	—	—
	ET1 ^k^	1.00 (0.87–1.38)	31	0.58 (0.53–0.62)	45	1.79 (1.53–2.09)	<0.001	<0.001	—	—	—
**Neonatal**	IL2	5.02 (4.39–5.76)	34	4.46 (2.62–5.44)	47	1.37 (1.11–1.71)	0.005	0.016	1.20 (0.99–1.45)	0.065	0.164
	IL6	69.79 (26.98–170.40)		86.13 (11.01–326.80)		1.03 (0.38–2.81)	0.951	0.951	1.01 (0.35–2.88)	0.987	0.987
	IL8	9.48 (4.77–28.07)		6.31 (4.57–13.89)		1.31 (0.82–2.11)	0.256	0.366	1.14 (0.65–1.97)	0.646	0.807
	TNFα	13.37 (10.54–14.91)		10.13 (8.49–11.58)		1.49 (1.22–1.82)	<0.001	<0.001	1.33 (1.14–1.55)	<0.001	0.003
	ET1	1.24 (0.96–1.58)		1.23 (0.88–1.39)		1.15 (0.96–1.38)	0.135	0.225	1.15 (0.92–1.44)	0.203	0.338

^a^ PE, preeclampsia; ^b^ IQR, interquartile range; ^c^ GMR, geometric mean ratio; ^d^ CI, confidence interval; ^e^ FDR, false discovery rate; ^f^ GA, gestational age; ^g^ IL2, interleukin 2; ^h^ IL6, interleukin 6; ^i^ IL8, interleukin 8; ^j^ TNFα, tumor necrosis factor α; ^k^ ET1, endothelin 1. Values are presented as median (IQR), with the analyzable sample size shown for each group. GMRs were obtained by exponentiating the PE-group coefficient from models fitted to natural-log-transformed biomarker concentrations and comparing the PE group with the control group. A GMR > 1 indicates a higher geometric mean concentration in the PE group; the null value is 1. Maternal models used HC3 robust standard errors, and neonatal models used pregnancy-clustered standard errors. Reported *p*-values are unadjusted, whereas q-values were obtained using the Benjamini–Hochberg FDR correction across the ten primary biomarker comparisons. GA-adjusted estimates represent secondary sensitivity analyses. Their five model-derived *p*-values constituted a separate multiple-testing family and were independently adjusted using the Benjamini–Hochberg procedure. They were not combined with the ten primary biomarker comparisons because the primary and gestational-age-adjusted models addressed different estimands. Maternal *p*-values were obtained from tests of the PE coefficient in log-linear models using HC3 heteroscedasticity-consistent standard errors. Primary neonatal *p*-values were obtained from log-linear models using pregnancy-clustered sandwich standard errors. Gestational-age-adjusted neonatal *p*-values were obtained from the corresponding pregnancy-clustered models including gestational age as a continuous covariate. *^§^* Two maternal IL6 concentrations (0.32 and 0.84 pg/mL) fell below the lowest non-zero calibration standard of 1 pg/mL and were therefore treated as non-quantifiable and excluded from all IL6-specific analyses. No fixed-value substitution or imputation was applied.

## Data Availability

The data presented in this study are available on request from the corresponding author due to privacy and ethical restrictions.

## Supplementary Materials

The following supporting information can be downloaded at: https://www.mdpi.com/article/10.3390/children13081039/s1, Table S1: This table presents the prespecified matching variables, their standardized mean differences (SMDs) before matching, immediately after matching, and in the final analytic cohort. This table also presents the model details; Table S2: Nominal concentrations of the eight calibration standards (pg/mL); Table S3: Post hoc exploratory Firth bias-reduced univariable logistic regression analyses of biomarker associations with severe preeclampsia, respiratory distress syndrome, and neonatal thrombocytopenia among mothers with preeclampsia and their neonates (PE-exposed groups); Table S4: Descriptive frequencies and biomarker concentrations among participants with rare maternal and neonatal outcomes. (A) Maternal biomarker concentrations among outcome-positive participants. (B) Neonatal biomarker concentrations among outcome-positive neonates.

## Abbreviations

The following abbreviations are used in this manuscript:

ACOG	American College of Obstetricians and Gynecologists
ALP	Alkaline phosphatase
ALT	Alanine aminotransferase
ANCOVA	Analysis of covariance
ART	Assisted reproductive technology
AST	Aspartate aminotransferase
ATT	Average treatment effect on the treated
AUC	Area under the curve
BMI	Body mass index
BPD	Bronchopulmonary dysplasia
CI	Confidence interval
COVID-19	Coronavirus disease 2019
CRP	C-reactive protein
CS	Cesarean section
ELISA	Enzyme-linked immunosorbent assay
ET1	Endothelin 1
FDR	False discovery rate
FM	Fibrin monomer
GA	Gestational age
gGT	Gamma-glutamyl transferase
GMR	Geometric mean ratio
GW	Gestational weeks
HC3	Heteroscedasticity-consistent covariance matrix estimator, type 3
HELLP	Hemolysis, elevated liver enzymes, and low platelet count
ICU	Intensive care unit
IL	Interleukin
IL2	Interleukin 2
IL6	Interleukin 6
IL8	Interleukin 8
IQR	Interquartile range
IUGR	Intrauterine growth restriction
IVH	Intraventricular hemorrhage
K3-EDTA	Tripotassium ethylenediaminetetraacetic acid
LDH	Lactate dehydrogenase
LLOQ	Lower limit of quantification
LMWH	Low-molecular-weight heparin
LOD	Limit of detection
MPV	Mean platelet volume
NA	Not applicable
NEC	Necrotizing enterocolitis
NICE	National Institute for Health and Care Excellence
NICHD	Eunice Kennedy Shriver National Institute of Child Health and Human Development
NICU	Neonatal intensive care unit
OR	Odds ratio
PE	Preeclampsia
PI	Pulsatility index
PLT	Platelet count
PMA	Postmenstrual age
PRES	Posterior reversible encephalopathy syndrome
RBC	Red blood cell
RDS	Respiratory distress syndrome
ROC	Receiver operating characteristic
ROP	Retinopathy of prematurity
ROTEM	Rotational thromboelastometry
SD	Standard deviation
SFMC	Soluble fibrin monomer complex
SGA	Small for gestational age
SLE	Systemic lupus erythematosus
SMD	Standardized mean difference
TNFα	Tumor necrosis factor α
VIF	Variance inflation factor
WBC	White blood cell

## References

[1] Marins L.R., Anizelli L.B., Romanowski M.D., Sarquis A.L. (2019). How Does Preeclampsia Affect Neonates? Highlights in the Disease’s Immunity. J. Matern.-Fetal Neonatal Med..

[2] Backes C.H., Markham K., Moorehead P., Cordero L., Nankervis C.A., Giannone P.J. (2011). Maternal Preeclampsia and Neonatal Outcomes. J. Pregnancy.

[3] Koulouraki S., Paschos V., Pervanidou P., Christopoulos P., Gerede A., Eleftheriades M. (2023). Short- and Long-Term Outcomes of Preeclampsia in Offspring: Review of the Literature. Children.

[4] Wilmink F.A., Reijnierse J., Reiss I.K.M., Steegers E.A.P., De Jonge R.C.J. (2019). Preeclampsia and Risk of Developing Bronchopulmonary Dysplasia in Very Preterm Neonates. Pregnancy Hypertens..

[5] Taylor R.N. (2015). Chesley’s Hypertensive Disorders in Pregnancy.

[6] Espinoza J., Vidaeff A., Pettker C.M., Simhan H. (2020). Gestational Hypertension and Preeclampsia. Obstet. Gynecol..

[7] Aaltonen R., Heikkinen T., Hakala K., Laine K., Alanen A. (2005). Transfer of Proinflammatory Cytokines Across Term Placenta. Obstet. Gynecol..

[8] Zaretsky M.V., Alexander J.M., Byrd W., Bawdon R.E. (2004). Transfer of Inflammatory Cytokines Across the Placenta. Obstet. Gynecol..

[9] Davidge S.T., Signorella A.P., Lykins D.L., Gilmour C.H., Roberts J.M. (1996). Evidence of Endothelial Activation and Endothelial Activators in Cord Blood of Infants of Preeclamptic Women. Am. J. Obstet. Gynecol..

[10] Døllner H., Vatten L., Linnebo I., Zanussi G.F., Lærdal Å., Austgulen R. (2001). Inflammatory Mediators in Umbilical Plasma from Neonates Who Develop Early-Onset Sepsis. Neonatology.

[11] Reis Machado J., Soave D.F., Da Silva M.V., De Menezes L.B., Etchebehere R.M., Monteiro M.L.G.D.R., Antônia Dos Reis M., Corrêa R.R.M., Celes M.R.N. (2014). Neonatal Sepsis and Inflammatory Mediators. Mediat. Inflamm..

[12] Bhandari V. (2014). Effective Biomarkers for Diagnosis of Neonatal Sepsis. J. Pediatr. Infect. Dis. Soc..

[13] Vasilescu D.I., Dan A.M., Dragomir I., Vasilescu S.L., Dumitru A.V., Dima V., Cîrstoiu M.M. (2025). Predicting Neonatal Morbidity and Correlations with Maternal and Neonatal Biomarkers in Connection with Fetal Inflammatory Response Syndrome in Premature Births. J. Clin. Med..

[14] Prashant A., Vishwanath P., Kulkarni P., Sathya Narayana P., Gowdara V., Nataraj S.M., Nagaraj R. (2013). Comparative Assessment of Cytokines and Other Inflammatory Markers for the Early Diagnosis of Neonatal Sepsis–A Case Control Study. PLoS ONE.

[15] Boskabadi H., Zakerihamidi M. (2018). Evaluate the Diagnosis of Neonatal Sepsis by Measuring Interleukins: A Systematic Review. Pediatr. Neonatol..

[16] Okazaki K., Nishida A., Kato M., Kozawa K., Uga N., Kimura H. (2006). Elevation of Cytokine Concentrations in Asphyxiated Neonates. Neonatology.

[17] Santana C., Guindeo M., González G., García-Muñoz F., Saavedra P., Doménech E. (2001). Cord Blood Levels of Cytokines as Predictors of Early Neonatal Sepsis. Acta Paediatr..

[18] Kontovazainitis C.-G., Gialamprinou D., Fleva A., Theodoridis T., Chatziioannidis I., Mitsiakou C., Banti A., Diamanti E., Mitsiakos G. (2025). Rotational Thromboelastometry (ROTEM) Hemostasis Profile in Pregnant Women with Preeclampsia and Their Offspring: An Observational Study. Diagnostics.

[19] Vidaeff A., Espinoza J., Simhan H., Pettker C.M. (2019). ACOG Practice Bulletin No. 203: Chronic Hypertension in Pregnancy. Obstet. Gynecol..

[20] NICE Guideline Hypertension in Pregnancy: Diagnosis and Management. https://www.nice.org.uk/guidance/ng133/resources/hypertension-in-pregnancy-diagnosis-and-management-pdf-66141717671365.

[21] Valensise H., Vasapollo B., Gagliardi G., Novelli G.P. (2008). Early and Late Preeclampsia: Two Different Maternal Hemodynamic States in the Latent Phase of the Disease. Hypertension.

[22] Robillard P., Dekker G., Scioscia M., Bonsante F., Iacobelli S., Boukerrou M., Hulsey T.C. (2020). Validation of the 34-week Gestation as Definition of Late Onset Preeclampsia: Testing Different Cutoffs from 30 to 37 Weeks on a Population-based Cohort of 1700 Preeclamptics. Acta Obs. Gynecol. Scand..

[23] Cremer M., Sallmon H., Kling P.J., Bührer C., Dame C. (2016). Thrombocytopenia and Platelet Transfusion in the Neonate. Semin. Fetal Neonatal Med..

[24] Wiedmeier S.E., Henry E., Sola-Visner M.C., Christensen R.D. (2009). Platelet Reference Ranges for Neonates, Defined Using Data from over 47,000 Patients in a Multihospital Healthcare System. J. Perinatol..

[25] Higgins R.D., Jobe A.H., Koso-Thomas M., Bancalari E., Viscardi R.M., Hartert T.V., Ryan R.M., Kallapur S.G., Steinhorn R.H., Konduri G.G. (2018). Bronchopulmonary Dysplasia: Executive Summary of a Workshop. J. Pediatr..

[26] Papile L.-A., Burstein J., Burstein R., Koffler H. (1978). Incidence and Evolution of Subependymal and Intraventricular Hemorrhage: A Study of Infants with Birth Weights Less than 1,500 Gm. J. Pediatr..

[27] Inder T., Perlman J., Volpe J., Volpe J.J. (2018). Preterm Intraventricular Hemorrhage/Posthemorrhagic Hydrocephalus. Volpe’s Neurology of the Newborn.

[28] Hand I.L., Shellhaas R.A., Milla S.S., Cummings J.J., Adams-Chapman I.S., Aucott S.W., Goldsmith J.P., Kaufman D.A., Martin C.R., Committee on Fetus and Newborn, Section on Neurology, Section on Radiology (2020). Routine Neuroimaging of the Preterm Brain. Pediatrics.

[29] Caplan M.S., Underwood M.A., Modi N., Patel R., Gordon P.V., Sylvester K.G., McElroy S., Manzoni P., Gephart S., Chwals W.J. (2019). Necrotizing Enterocolitis: Using Regulatory Science and Drug Development to Improve Outcomes. J. Pediatr..

[30] Walsh M.C., Kliegman R.M. (1986). Necrotizing Enterocolitis: Treatment Based on Staging Criteria. Pediatr. Clin. N. Am..

[31] Chiang M.F., Quinn G.E., Fielder A.R., Ostmo S.R., Paul Chan R.V., Berrocal A., Binenbaum G., Blair M., Peter Campbell J., Capone A. (2021). International Classification of Retinopathy of Prematurity, Third Edition. Ophthalmology.

[32] Fierson W.M., Chiang M.F., Good W., Phelps D., Reynolds J., Robbins S.L., American Academy of Pediatrics Section on Ophthalmology, American Academy of Ophthalmology, American Association for Pediatric Ophthalmology and Strabismus, American Association of Certified Orthoptists (2018). Screening Examination of Premature Infants for Retinopathy of Prematurity. Pediatrics.

[33] Sweet D.G., Carnielli V.P., Greisen G., Hallman M., Klebermass-Schrehof K., Ozek E., Te Pas A., Plavka R., Roehr C.C., Saugstad O.D. (2023). European Consensus Guidelines on the Management of Respiratory Distress Syndrome: 2022 Update. Neonatology.

[34] Aggarwal R., Jain A.K., Mittal P., Kohli M., Jawanjal P., Rath G. (2019). Association of Pro- and Anti-Inflammatory Cytokines in Preeclampsia. J. Clin. Lab. Anal..

[35] Karakus S., Bozoklu Akkar O., Yildiz C., Sancakdar E., Cetin M., Cetin A. (2016). Serum Levels of ET-1, M30, and Angiopoietins-1 and -2 in HELLP Syndrome and Preeclampsia Compared to Controls. Arch. Gynecol. Obstet..

[36] El Sayed M., Sherif L., Said R.N., El-Wakkad A.S.E., El-Refay A., Aly H. (2011). Endothelin-1 and L-Arginine in Preterm Infants with Respiratory Distress. Am. J. Perinatol..

[37] Keene O.N. (1995). The Log Transformation Is Special. Stat. Med..

[38] Benjamini Y., Hochberg Y. (1995). Controlling the False Discovery Rate: A Practical and Powerful Approach to Multiple Testing. J. R. Stat. Soc. Ser. B Stat. Methodol..

[39] Firth D. (1993). Bias Reduction of Maximum Likelihood Estimates. Biometrika.

[40] Heinze G., Schemper M. (2002). A Solution to the Problem of Separation in Logistic Regression. Stat. Med..

[41] Al-Othman S., Omu A.E., Diejomaoh F.M.E., Al-Yatama M., Al-Qattan F. (2001). Differential Levels of Interleukin 6 in Maternal and Cord Sera and Placenta in Women with Pre-Eclampsia. Gynecol. Obs. Investig..

[42] Tosun M., Celik H., Avci B., Yavuz E., Alper T., Malatyalioğlu E. (2010). Maternal and Umbilical Serum Levels of Interleukin-6, Interleukin-8, and Tumor Necrosis Factor-α in Normal Pregnancies and in Pregnancies Complicated by Preeclampsia. J. Matern. -Fetal Neonatal Med..

[43] Guillemette L., Lacroix M., Allard C., Patenaude J., Battista M.-C., Doyon M., Moreau J., Ménard J., Ardilouze J.-L., Perron P. (2015). Preeclampsia Is Associated with an Increased Pro-Inflammatory Profile in Newborns. J. Reprod. Immunol..

[44] Nguyen P.N., Ramos A. (2026). Associated Factors Relating to the Success of Labor Induction among Pregnant Women Diagnosed with Fetal Intrauterine Growth Restriction: A Retrospective Cohort Study from France. AJP Rep..

[45] Tsao P.-N., Teng R.-J., Chou H.-C., Tsou K.-I. (2002). The Thrombopoietin Level in the Cord Blood in Premature Infants Born to Mothers with Pregnancy-Induced Hypertension. Neonatology.

[46] Bayoumi M.A.A., Ali A.A.H., Hamad S.G., Ali A.A.M., Elmalik E.E., Elkalaf M.M.I.R., Moustafa B.A.A., Shaltout D.A.D.A., Chandra P., Langtree L.J. (2020). Effect of Maternal Preeclampsia on Hematological Profile of Newborns in Qatar. BioMed Res. Int..

[47] Ramesh Bhat Y., Cherian C.S. (2008). Neonatal Thrombocytopenia Associated with Maternal Pregnancy Induced Hypertension. Indian. J. Pediatr..

[48] Roberts I., Murray N.A. (2003). Neonatal Thrombocytopenia: Causes and Management. Arch. Dis. Child. Fetal Neonatal Ed..

[49] Beiner M.E., Simchen M.J., Sivan E., Chetrit A., Kuint J., Schiff E. (2003). Risk Factors for Neonatal Thrombocytopenia in Preterm Infants. Am. J. Perinatol..

[50] Baschat A.A., Gembruch U., Reiss I., Gortner L., Weiner C.P., Harman C.R. (2000). Absent Umbilical Artery End-Diastolic Velocity in Growth-Restricted Fetuses: A Risk Factor for Neonatal Thrombocytopenia. Obstet. Gynecol..

[51] Zook K.J., Mackley A.B., Kern J., Paul D.A. (2009). Hematologic Effects of Placental Pathology on Very Low Birthweight Infants Born to Mothers with Preeclampsia. J. Perinatol..

[52] Jobe A.H., Bancalari E. (2001). Bronchopulmonary Dysplasia. Am. J. Respir. Crit. Care Med..

[53] Gien J., Kinsella J.P. (2011). Pathogenesis and Treatment of Bronchopulmonary Dysplasia. Curr. Opin. Pediatr..

[54] Dankhara N., Holla I., Ramarao S., Kalikkot Thekkeveedu R. (2023). Bronchopulmonary Dysplasia: Pathogenesis and Pathophysiology. J. Clin. Med..

[55] Pan J., Zhan C., Yuan T., Wang W., Shen Y., Sun Y., Wu T., Gu W., Chen L., Yu H. (2018). Effects and Molecular Mechanisms of Intrauterine Infection/Inflammation on Lung Development. Respir. Res..

[56] Zhang Z., Wu W., Hou L., Jiang J., Wan W., Li Z. (2021). Cytokines and Exhaled Nitric Oxide Are Risk Factors in Preterm Infants for Bronchopulmonary Dysplasia. BioMed Res. Int..

[57] Li H., Wang G., Lin S., Wang C., Zha J. (2019). Loss of Interleukin-6 Enhances the Inflammatory Response Associated with Hyperoxia-induced Lung Injury in Neonatal Mice. Exp. Ther. Med..

[58] Ehrhardt H., Pritzke T., Oak P., Kossert M., Biebach L., Förster K., Koschlig M., Alvira C.M., Hilgendorff A. (2016). Absence of TNF-α Enhances Inflammatory Response in the Newborn Lung Undergoing Mechanical Ventilation. Am. J. Physiol.-Lung Cell. Mol. Physiol..

[59] Mohamed H.A.E., Yosef Y.M., Mohamed N.A.E., Alsyed A.A.A. (2022). Endothelin 1 as A Predictor Marker for Bronchopulmonary Dysplasia in Preterm Neonates with Respiratory Distress Syndrome. Egypt. J. Hosp. Med..

[60] Rivera L., Siddaiah R., Oji-Mmuo C., Silveyra G.R., Silveyra P. (2016). Biomarkers for Bronchopulmonary Dysplasia in the Preterm Infant. Front. Pediatr..

[61] Kassal R., Anwar M., Kashlan F., Smulian J., Hiatt M., Hegyi T. (2005). Umbilical Vein Interleukin-6 Levels in Very Low Birth Weight Infants Developing Intraventricular Hemorrhage. Brain Dev..

[62] Heep A., Behrendt D., Nitsch P., Fimmers R., Bartmann P., Dembinski J. (2003). Increased Serum Levels of Interleukin 6 Are Associated with Severe Intraventricular Haemorrhage in Extremely Premature Infants. Arch. Dis. Child. Fetal Neonatal Ed..

[63] Abdel Raheem Y.F., Mohamad J.I., Mohammed E.R., Ibrahiem R.M., Yousef A.M., Ali Ahmed A.M., Shalaby A.M. (2026). Cord Blood Cytokines as Early Predictors for Retinopathy of Prematurity. J. Neonatal-Perinat. Med..

[64] Meister A.L., Gardner F.C., Browning K.N., Travagli R.A., Palmer C., Doheny K.K. (2021). Vagal Tone and Proinflammatory Cytokines Predict Feeding Intolerance and Necrotizing Enterocolitis Risk. Adv. Neonatal Care.

[65] Gitto E., Reiter R.J., Amodio A., Romeo C., Cuzzocrea E., Sabatino G., Buonocore G., Cordaro V., Trimarchi G., Barberi I. (2004). Early Indicators of Chronic Lung Disease in Preterm Infants with Respiratory Distress Syndrome and Their Inhibition by Melatonin. J. Pineal Res..

[66] Gitto E., Reiter R.J., Cordaro S.P., La Rosa M., Chiurazzi P., Trimarchi G., Gitto P., Calabró M.P., Barberi I. (2004). Oxidative and Inflammatory Parameters in Respiratory Distress Syndrome of Preterm Newborns: Beneficial Effects of Melatonin. Am. J. Perinatol..

[67] Kuo C.Y., Chou Y.H., Lien R., Yang P.H. (2001). Study of Plasma Endothelin-1 Concentrations in Taiwanese Neonates with Respiratory Distress. Chang Gung Med. J..

[68] Vuong A.D.B., Tran N.H., Pham T.H., Le H.A.M., Nguyen P.N. (2025). Soluble Fibrin Monomer Complex and D-Dimer Concentrations Between Patients at Low and High Risk of Venous Thromboembolism Before Delivery According to RCOG Score Assessment: An Observational Study Among 100 Third-Trimester Vietnamese Pregnancies. J. Clin. Med..

[69] Chiesa C., Pacifico L., Natale F., Hofer N., Osborn J.F., Resch B. (2015). Fetal and Early Neonatal Interleukin-6 Response. Cytokine.

[70] Zhou X., Fragala M.S., McElhaney J.E., Kuchel G.A. (2010). Conceptual and Methodological Issues Relevant to Cytokine and Inflammatory Marker Measurements in Clinical Research. Curr. Opin. Clin. Nutr. Metab. Care.

