# Supplementary material for: Maternal and Early Neonatal Serum Cytokine and Endothelin-1 Concentrations in Preeclampsia: A Single-Center Observational Cohort Study
Source: Children (Basel). 2026 Aug 4;13(8):1039. doi: 10.3390/children13081039 (PMC13510692; doi:10.3390/children13081039)
Supplement: Supplementary file 1 [file children-13-01039-s001.zip › children-4450284-supplementary.pdf]

Table S1. This table presents the prespecified matching variables, their standardized mean differences (SMDs) before matching, immediately after matching, and in the final analytic cohort. This table also presents the model details.

Covariate	Before matching 47 PE[a] / 1413 Controls	After nearest-neighbor matching 1:1 47 PE / 47 Controls SMD[b]s	Final analytic cohort 31 PE / 45 Controls SMDs
**Maternal age**	**+0.311**	−0.010	**+0.173**
**GA[c] at recruitment**	**+0.173**	+0.072	**0.101**
**Pre-pregnancy BMI[d]**	**+0.369**	**+0.152**	**+0.145**
**Maternal age ≥40 years**	+0.087	−0.096	+0.053
**Preeclampsia in a previous pregnancy**	**+0.345**	0.000	**+0.102**
**Aspirin**	**+0.567**	0.000	**+0.308**
**Low-molecular-weight heparin**	+0.321	+0.066	+0.065
**Progesterone**	**−0.275**	0.000	**−0.129**
**Antenatal corticosteroids**	**+0.412**	+0.069	**+0.118**

Model Details

**Algorithm / objective**	Greedy nearest-neighbor assignment on the propensity score; PE units processed from largest to smallest propensity score.
**Absolute PS distance: total / mean / median / maximum**	0.5891 / 0.0125 / 0.0114 / 0.1064
**Maximum absolute logit-PS[e] distance**	0.4340
**Covariates with ∣SMD∣ ≤0.10 after matching**	8/9
**Covariates with 0.10 < ∣SMD∣ ≤ 0.20 after matching**	1/9
**Covariates with ∣SMD∣ >0.20 after matching**	0/9
**Caliper**	no caliper width or scale applied
**Replacement**	No
**Estimand**	ATT[f]

**Mean |SMD| immediately after matching**	0.052
**Maximum |SMD| immediately after matching**	0.152

[a]PE: Preeclampsia; [b]SMD: Standardized Mean Difference; [c]GA: gestational Age; [d]BMI: Body Mass Index; [e]PS: Propensity Score; [f]ATT: Average Treatment effect on the Treated

Signed SMDs are presented; absolute values were used to assess balance. Under the ATT estimand, continuous and binary covariates were standardized using the corresponding pre-matching PE-group standard deviation, and the same standardization factor was retained across all three stages.

Table S2. Nominal concentrations of the eight calibration standards (pg/mL).

Analyte	Calibration-standard concentrations, excluding the blank (pg/mL)	Calibration range (pg/mL)	Archived R^2
IL2[a]	1.000; 2.743; 8.230; 24.691; 74.074; 222.222; 666.667; 2,000	1–2,000	0.999991
IL6[b]	1.000; 4.115; 12.346; 37.037; 111.111; 333.333; 1,000; 3,000	1–3,000	0.999939
IL8[c]	1.000; 2.743; 8.230; 24.691; 74.074; 222.222; 666.667; 2,000	1–2,000	0.999998
TNFα[d]	1.000; 2.743; 8.230; 24.691; 74.074; 222.222; 666.667; 2,000	1–2,000	0.999973

[a]IL2, interleukin 2; [b]IL6, interleukin 6; [c]IL8, interleukin 8; [d]TNFα, tumor necrosis factor α

Table S3. Post hoc exploratory Firth bias-reduced univariable logistic regression analyses of biomarker associations with severe preeclampsia, respiratory distress syndrome, and neonatal thrombocytopenia among mothers with preeclampsia and their neonates (PE-exposed groups).

Outcome	Biomarker source	Biomarker	Active n/events	OR[a] per twofold increase (95% profile CI[b])	Raw p-value	FDR[c] q-value
**Severe PE[d]**	Maternal	IL2[f]	31/11	3.07 (0.62–21.26)	0.176	0.420
	Maternal	IL6[g]	30/10	1.29 (0.86–2.03)	0.211	0.420
	Maternal	IL8[h]	31/11	1.78 (0.66–6.53)	0.264	0.440
	Maternal	TNFα[i]	31/11	2.37 (0.16–41.75)	0.526	0.619
	Maternal	ET1[j]	31/11	2.41 (0.58–12.64)	0.231	0.420
**RDS[e]**	Neonatal	IL2	31/13	2.86 (1.21–8.91)	0.016	0.154
	Neonatal	IL6	31/13	0.93 (0.76–1.14)	0.496	0.619
	Neonatal	IL8	31/13	1.27 (0.86–1.89)	0.229	0.420
	Neonatal	TNFα	31/13	2.02 (0.99–5.62)	0.054	0.154
	Neonatal	ET1	31/13	0.99 (0.36–2.84)	0.986	0.986
**Neonatal thrombocytopenia**	Maternal	IL2	30/5	5.04 (0.994–35.69)	0.0509	0.154
	Maternal	IL6	29/4	0.89 (0.45–1.51)	0.680	0.716
	Maternal	IL8	30/5	1.54 (0.57–3.63)	0.350	0.533
	Maternal	TNFα	30/5	1.97 (0.11–34.04)	0.636	0.707
	Maternal	ET1	30/5	3.84 (0.999–19.21)	0.0501	0.154
	Neonatal	IL2	30/5	3.24 (1.03–11.92)	0.044	0.154
	Neonatal	IL6	30/5	1.14 (0.85–1.61)	0.404	0.539
	Neonatal	IL8	30/5	1.29 (0.73–2.29)	0.373	0.533
	Neonatal	TNFα	30/5	2.16 (1.01–5.92)	0.048	0.154
	Neonatal	ET1	30/5	15.67 (2.00–237.04)	0.006	0.120

[a]OR: Odds Ratio; [b]CI: Confidence Interval; [c]FDR: False Discovery Rate; [d]PE: Preeclampsia; [e]RDS: Respiratory Distress Syndrome; [f]IL2: Interleukin 2; [g]IL6: Interleukin 6; [h]IL8: Interleukin 8; [i]TNFα: Tumor Necrosis Factor α; [j]ET1: Endothelin 1

Statistical analysis. Biomarker concentrations were $\log_2$-transformed; therefore, odds ratios represent the change in outcome odds associated with a twofold higher biomarker concentration. Each biomarker was assessed in a separate univariable Firth bias-reduced logistic regression model. Profile penalized-likelihood 95% confidence intervals and penalized likelihood-ratio p-values are reported. To avoid within-pregnancy dependence, only the first enrolled neonate from each pregnancy was retained according to the study identification order. Consequently, each pregnancy contributed a single neonatal observation, and no correction for within-pregnancy clustering was required in these post hoc Firth models. Severe-PE analyses were restricted to 31 PE mothers (11 events) except for the maternal IL6 model, which included 30 mothers (10 events) after exclusion of one non-quantifiable IL6 concentration. RDS analyses included 31 neonates (13 events), and neonatal-thrombocytopenia analyses included 30 neonates (5 events); one neonate was excluded because the platelet count at birth was unavailable. The corresponding maternal IL6 model included 29 pregnancies/mother–neonate pairs (4 events) after the additional exclusion of one non-quantifiable maternal IL6 concentration.

The reported active sample size therefore represents the number of contributing pregnancies. The 20 associations were estimated using separate univariable Firth models. The Benjamini–Hochberg procedure was then applied jointly to the 20 resulting penalized likelihood-ratio p-values. The grouping of rows by outcome and biomarker source is solely for ease of presentation and does not represent separate adjustment sets. None of the associations remained statistically significant after FDR correction. These analyses are strictly hypothesis-generating and should not be interpreted as predictive models.

Two maternal IL6 concentrations (0.32 and 0.84 pg/mL) fell below the lowest non-zero calibration standard of 1 pg/mL and were therefore treated as non-quantifiable and excluded from all IL6-specific analyses. No fixed-value substitution or imputation was applied.

Table S4. Descriptive frequencies and biomarker concentrations among participants with rare maternal and neonatal outcomes.

Panel A. Maternal biomarker concentrations among outcome-positive participants

Outcome	PE[a] group n/N (%)	Control group n/N (%)	Active n	IL2[b] median (IQR[k])	IL6[c] median (IQR)	IL8[d] median (IQR)	TNFα[e] median (IQR)	ET1[f] median (IQR)
**Maternal platelet transfusion**	5/31 (16.1%)	0/45 (0%)	5	5.92 (4.67–6.11)	7.69 (5.41–25.46)	3.98 (3.72–4.76)	10.79 (10.79–12.05)	0.96 (0.91–1.37)
**Maternal postpartum thrombocytopenia**	3/31 (9.7%)	0/45 (0%)	3	4.67 (4.00–5.39)	7.69 (6.55–16.58)	3.72 (3.53–4.33)	10.79 (10.79–11.70)	0.96 (0.93–1.56)
**Neonatal thrombocytopenia at birth (paired maternal biomarkers)**	5/33 (15.2%)	1/47 (2.1%)	5	5.92 (4.39–6.11)	4.10 (3.57–5.15)	3.98 (3.85–4.76)	9.31 (8.21–12.05)	1.01 (0.75–1.37)

Panel B. Neonatal biomarker concentrations among outcome-positive neonates

Outcome	PE group n/N (%)	Control group n/N (%)	Active n	IL2 median (IQR)	IL6 median (IQR)	IL8 median (IQR)	TNFα median (IQR)	ET1 median (IQR)
**Neonatal thrombocytopenia at birth**	5/33 (15.2%)	1/47 (2.1%)	6	5.95 (5.12–6.25)	182.22 (36.10–496.19)	10.87 (8.11–22.84)	13.45 (10.82–13.70)	1.70 (1.49–2.05)
**BPD[g]**	5/11 (45.5%)	2/11 (18.2%)	7	5.36 (4.81–8.87)	76.53 (54.52–208.40)	13.25 (5.28–27.39)	13.75 (11.56–22.83)	1.30 (1.05–1.64)
**IVH[h]**	2/34 (5.9%)	2/47 (4.3%)	4	4.81 (4.80–10.21)	73.17 (62.80–88.98)	43.99 (35.02–47.14)	12.99 (11.72–92.34)	1.58 (1.09–1.98)
**NEC[i] (modified Bell stage ≥ II)**	2/34 (5.9%)	1/47 (2.1%)	3	5.53 (5.17–5.89)	208.40 (156.59–260.20)	28.62 (20.94–36.30)	13.37 (13.18–13.56)	1.64 (1.61–1.67)
**ROP[j] screening-eligible neonates**	3/13 (23.1%)	1/15 (6.7%)	4	6.25 (5.32–10.93)	76.53 (64.48–194.26)	13.25 (8.94–19.64)	15.40 (14.57–93.55)	1.70 (1.29–2.03)

[a]PE: Preeclampsia; [b]IL2: Interleukin 2; [c]IL6: Interleukin 6; [d]IL8: Interleukin 8; [e]TNFα: Tumor Necrosis Factor α; [f]ET1: Endothelin 1; [g]BPD: Bronchopulmonary Dysplasia; [h]IVH: Intraventricular Hemorrhage; [i]NEC: Necrotizing Enterocolitis; [j]ROP: Retinopathy of Prematurity; [k]IQR: Interquartile Range

Values are n/N (%) for clinical outcome frequencies and median (IQR) in pg/mL for biomarker concentrations among outcome-positive participants with complete measurements for all five biomarkers in the relevant panel. The biomarker n may therefore be smaller than the clinical event count. All maternal transfusions were platelet transfusions. BPD denominators were restricted to neonates born at <32 gestational weeks (GW) who survived to and were assessed at 36^{+0} weeks' postmenstrual age (PMA). One eligible neonate in each group died before assessment and was excluded; IVH denominators include all enrolled neonates. All enrolled neonates underwent at least one cranial ultrasonographic examination within the first 24 hours of life as part of the study assessment and a concurrent neonatal protocol. Neonates born at <32 GW or with birthweight <1500 g underwent routine serial cranial ultrasonography on postnatal days 1, 3, and 7. Additional examinations were performed when the initial examination was abnormal or when clinically indicated. The IVH cases were grade II and grade III in the PE-exposed group and grade II and grade II in the control group; ROP denominators include neonates born at <32 gestational weeks or with birthweight <1500 g. All neonates, including both members of twin pairs, were retained in these descriptive summaries. No inferential comparisons are presented in this table. Exploratory Firth results for neonatal thrombocytopenia are reported separately in Table S3.

Two maternal IL6 concentrations (0.32 and 0.84 pg/mL) fell below the lowest non-zero calibration standard of 1 pg/mL and were therefore treated as non-quantifiable and excluded from all IL6-specific analyses. No fixed-value substitution or imputation was applied. For the paired maternal-biomarker summary of neonatal thrombocytopenia, one outcome-positive maternal observation was excluded because its IL6 concentration was below the lowest non-zero calibration standard. Therefore, five outcome-positive participants had complete quantifiable measurements for all five maternal biomarkers.
